# Cardioprotective properties of OMT-28, a synthetic analog of omega-3 epoxyeicosanoids

**DOI:** 10.1016/j.jbc.2024.107372

**Published:** 2024-05-15

**Authors:** Joshua Kranrod, Anne Konkel, Robert Valencia, Ahmed M. Darwesh, Robert Fischer, Wolf-Hagen Schunck, John M. Seubert

**Affiliations:** 1 Faculty of Pharmacy and Pharmaceutical Sciences, College of Health Sciences, University of Alberta, Edmonton, Alberta, Canada; 2 Cardiovascular Research Institute, University of Alberta, Edmonton, Alberta, Canada; 3 OMEICOS Therapeutics GmbH, Berlin, Germany; 4 Faculty of Medicine and Dentistry, Department of Pharmacology, College of Health Sciences, University of Alberta, Edmonton, Alberta, Canada; 5 Max Delbrueck Center for Molecular Medicine, Berlin, Germany

**Keywords:** OMT-28, cardioprotection, analog, 17,18-EEQ, oxylipin, eicosanoid

## Abstract

OMT-28 is a metabolically robust small molecule developed to mimic the structure and function of omega-3 epoxyeicosanoids. However, it remained unknown to what extent OMT-28 also shares the cardioprotective and anti-inflammatory properties of its natural counterparts. To address this question, we analyzed the ability of OMT-28 to ameliorate hypoxia/reoxygenation (HR)-injury and lipopolysaccharide (LPS)-induced endotoxemia in cultured cardiomyocytes. Moreover, we investigated the potential of OMT-28 to limit functional damage and inflammasome activation in isolated perfused mouse hearts subjected to ischemia/reperfusion (IR) injury. In the HR model, OMT-28 (1 μM) treatment largely preserved cell viability (about 75 *versus* 40% with the vehicle) and mitochondrial function as indicated by the maintenance of NAD+/NADH-, ADP/ATP-, and respiratory control ratios. Moreover, OMT-28 blocked the HR-induced production of mitochondrial reactive oxygen species. Pharmacological inhibition experiments suggested that Gαi, PI3K, PPARα, and Sirt1 are essential components of the OMT-28-mediated pro-survival pathway. Counteracting inflammatory injury of cardiomyocytes, OMT-28 (1 μM) reduced LPS-induced increases in TNFα protein (by about 85% *versus* vehicle) and NF-κB DNA binding (by about 70% *versus* vehicle). In the *ex vivo* model, OMT-28 improved post-IR myocardial function recovery to reach about 40% of the baseline value compared to less than 20% with the vehicle. Furthermore, OMT-28 (1 μM) limited IR-induced NLRP3 inflammasome activation similarly to a direct NLRP3 inhibitor (MCC950). Overall, this study demonstrates that OMT-28 possesses potent cardio-protective and anti-inflammatory properties supporting the hypothesis that extending the bioavailability of omega-3 epoxyeicosanoids may improve their prospects as therapeutic agents.

N-3 polyunsaturated fatty acids (n-3 PUFAs), eicosapentaenoic acid (EPA) and docosahexaenoic acid (DHA), can be endogenously metabolized into bioactive epoxylipids by cytochrome P450 enzymes (1, 2). Numerous studies have suggested that increased consumption of n-3 PUFAs decreases the morbidity and mortality related to cardiovascular disease. Dietary supplementation with EPA and DHA has resulted in increased plasma and tissue levels of the epoxylipids, 17,18-epoxyeicosatetraenoic acid (17,18-EEQ), and 19,20-epoxydocosapentaenoic acid (19,20-EDP) (1, 3, 4). These n-3-derived FA epoxides (n-3 EpFA) display pleiotropic effects including cardioprotective (5, 6), antihypertensive (7), anti-inflammatory (8, 9, 10), and antifibrotic (11) properties that may contribute to the beneficial effects of n-3 LC-PUFAs in cardiovascular disease (2). Preclinical animal studies demonstrate synthetic analogs of n-3 epoxyeicosanoids can ameliorate ischemia-reperfusion injury (12, 13), laser-induced choroidal neovascularization (8), and LPS-induced endotoxemia (14).

EPA and DHA-derived epoxylipids undergo rapid autoxidation, membrane incorporation, and enzymatic metabolism by cyclooxygenases, lipoxygenases, and epoxide hydrolases under normal physiological conditions (2). To overcome these limitations, chemically and metabolically robust synthetic analogs of 17,18-EEQ have been developed (15, 16). Improved drug-like properties were achieved by modifying the chemical structure of natural metabolites while preserving their effects on cardiomyocyte contraction (Fig. 1). Among the 17,18-EEQ analogs synthesized, OMT-28, showed superior metabolic stability, high oral bioavailability, and favorable pharmacokinetics (15). OMT-28 is currently investigated in Phase II clinical studies in patients with cardiovascular and mitochondrial disease by OMEICOS Therapeutics (https://omeicos.com/).

**Figure 1:**
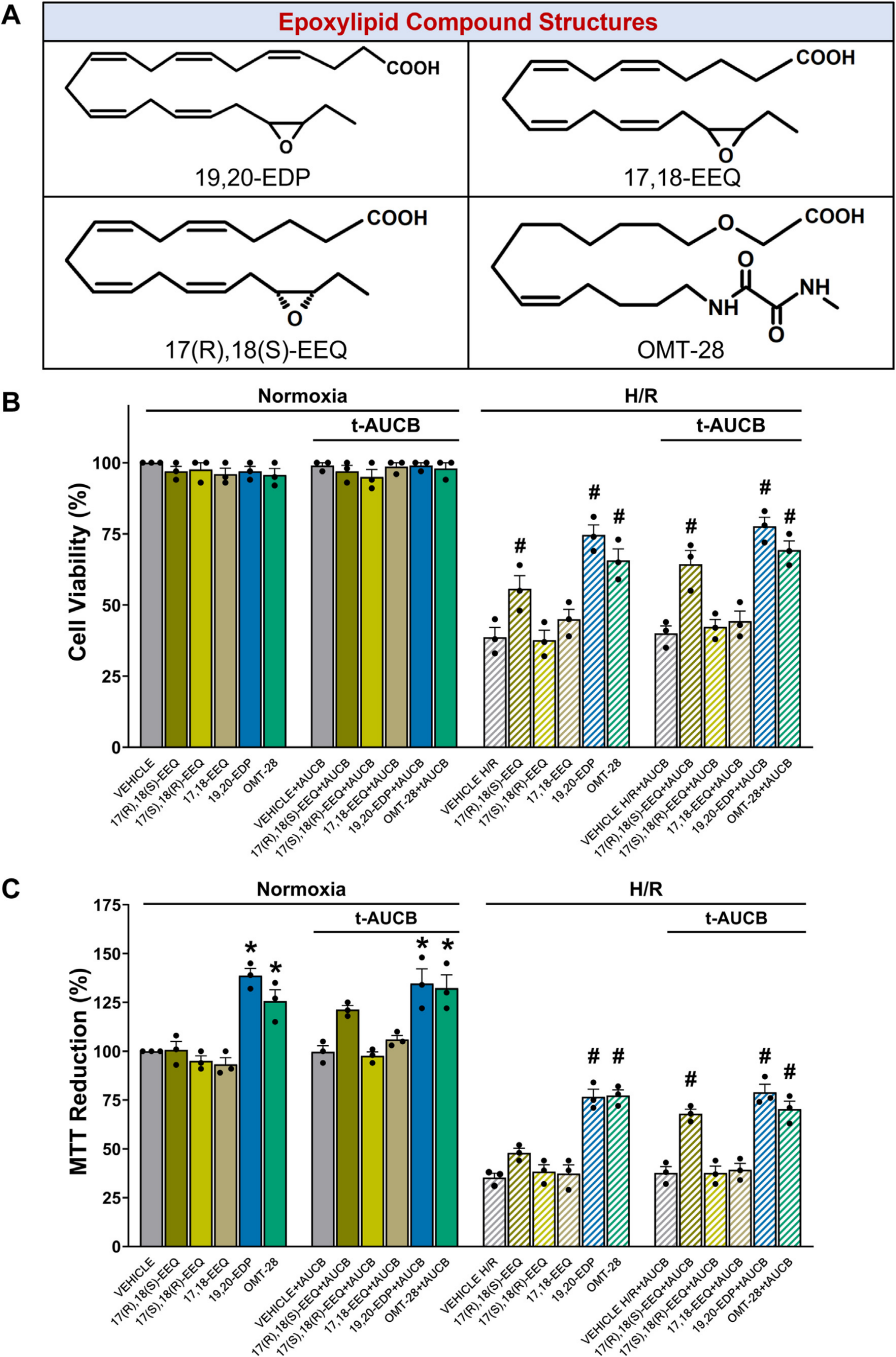
**OMT-28 protects HL-1 cells against HR injury.***A*, structure of 19,20-EDP, 17,18-EEQ and OMT-28. HL-1 cells treated with 1 μM either 17(R),18(S)-EEQ, 17(S),18(R)-EEQ, racemic 17,18-EEQ, 19,20-EDP, or OMT-28 with or without *t*AUCB (1 μM) and subjected to normoxia or hypoxia-reoxygenation. Colorimetric analysis of EpFA-induced changes on cell viability and mitochondrial oxidative metabolism following HR injury. *B*, WST-8 (CCK-8) cell viability assay. *C*, cell proliferation (MTT). Values represent mean ± SEM, data were obtained by analyzing responses of three independent cell preparations and using at least three technical replicas. *p* < 0.05 statistically significant, one-way ANOVA, Bonferroni *post hoc* test, ∗ *versus* vehicle control normoxia; # *versus* vehicle control HR.

In the present study, we investigate the cardioprotective properties of our novel synthetic analog compared to a naturally occurring epoxylipid, 19,20-EDP. To this end, we tested OMT-28 for its ability to protect cardiac cells against HR- and LPS-induced injury. Moreover, we analyzed OMT-28 effects on ischemia/reperfusion injury using isolated perfused hearts.

## Results

### OMT-28 protects cardiomyocytes against hypoxia/reoxygenation-induced loss of cell viability and mitochondrial function

#### Cytoprotective effects of OMT-28 in cardiomyocytes subjected to hypoxia-reoxygenation injury

First, we assessed cytoprotection by OMT-28 and various EpFAs using a HL-1 murine cardiac cell hypoxia-reoxygenation (HR) injury model. In parallel, we compared OMT-28 to its endogenous analog, 17,18-EEQ. 17,18-EEQ was added either as the R,S-enantiomer, the S,R-enantiomer, or as the racemic mixture of 17,18-EEQ. OMT-28 was also compared to another cardioprotective endogenous n-3 PUFA epoxide, 19,20-EDP (12, 17). All compounds were also co-treated with *t*AUCB to assess whether sEH-mediated hydrolysis inhibits the beneficial effects of epoxyeicosanoid treatment.

Using the CCK-8 assay to measure cell viability, we observed no cytotoxic effects for any compound under normoxic conditions (Fig. 1*B*). HR injury alone reduced cell viability to 40% of normoxic control (vehicle HR *versus* vehicle normoxia in Fig. 1*B*). Both OMT-28 and 17(R),18(S)-EEQ significantly limited cell loss compared to HR-vehicle control (1.7-fold and 1.4-fold respectively), but 17(S),18(R)-EEQ or racemic 17,18-EEQ failed to protect the cells. Cells incubated with 19,20-EDP significantly increased metabolic activity after HR-injury compared to HR control (1.9-fold higher). Consistent with previous studies (18, 19), *t*AUCB co-treatment moderately improved 17(R),18(S)-EEQ cytoprotection (to 1.6-fold HR control), presumably because limiting sEH-mediated hydrolysis improves the long-term stability of this compound. Notably, sEH inhibition did not improve the efficacy of 17(S),18(R)-EEQ or racemic 17,18-EEQ, suggesting that the R,S-enantiomer is likely the dominant bioactive molecule. In contrast, *t*AUCB co-treatment did not enhance 19,20-EDP-mediated cytoprotection, despite 19,20-EDP possessing an sEH-labile epoxide group. Inhibition of sEH did not affect OMT-28 efficiency, as expected.

Samokhvalov *et al.* (17) previously identified the capacity of 19,20-EDP to protect cardiac oxidative metabolism against hypoxic insult. Thus, we assessed OMT-28 and other endogenous n-3 EpFAs for their ability to protect mitochondrial oxidative metabolism using an MTT reduction assay (Fig. 1*C*). Interestingly, we observed that both 19,20-EDP and OMT-28 significantly increased MTT reduction already at normoxic conditions (1.4- and 1.3-fold of normoxic control, respectively), and both compounds also rescued MTT reduction in HR injury (both 2.2-fold of HR control). Additionally, while 17(R),18(S)-EEQ treatment alone could not significantly increase MTT reduction above HR control levels, 17(R),18(S)-EEQ plus *t*AUCB treatment significantly restored MTT reduction in HR (1.9-fold HR control). *t*AUCB co-treatment did not enhance MTT reduction for any other compound.

Overall, these data suggest that OMT-28 possesses potent cytoprotective effects against HR injury in HL-1 cardiac cells similar to endogenous EpFA comparators at a given concentration of 1 μM.

#### PI3Kα, Gα_i_, PPARα, and SIRT1 are involved in OMT-28-mediated cytoprotection against HR injury

To further investigate cytoprotective mechanisms of OMT-28 in cardiac cells following HR injury, we used selective pharmacological inhibitors to conduct a limited screen of putative signaling pathways involved in the OMT-28 response; specifically, PI3K (20, 21), G protein subunits (22), sarcolemmal ATP-dependent potassium (sK_ATP_) channel (23), PPARs (24, 25, 26), and SIRT1 (17) have all been associated with endogenous n-3/n-6 PUFA epoxide activity. Additionally, because OMT-28 significantly outperformed endogenous 17,18-EEQ enantiomers in our HL-1 HR injury model, we used 19,20-EDP as the sole comparator molecule to OMT-28 for subsequent *in vitro* experiments. We also utilized primary neonatal rat cardiomyocytes (NRCMs) for our remaining *in vitro* HR injury experiments, as they are more phenotypically like terminally differentiated *in vivo* cardiomyocytes (27).

**Figure 2:**
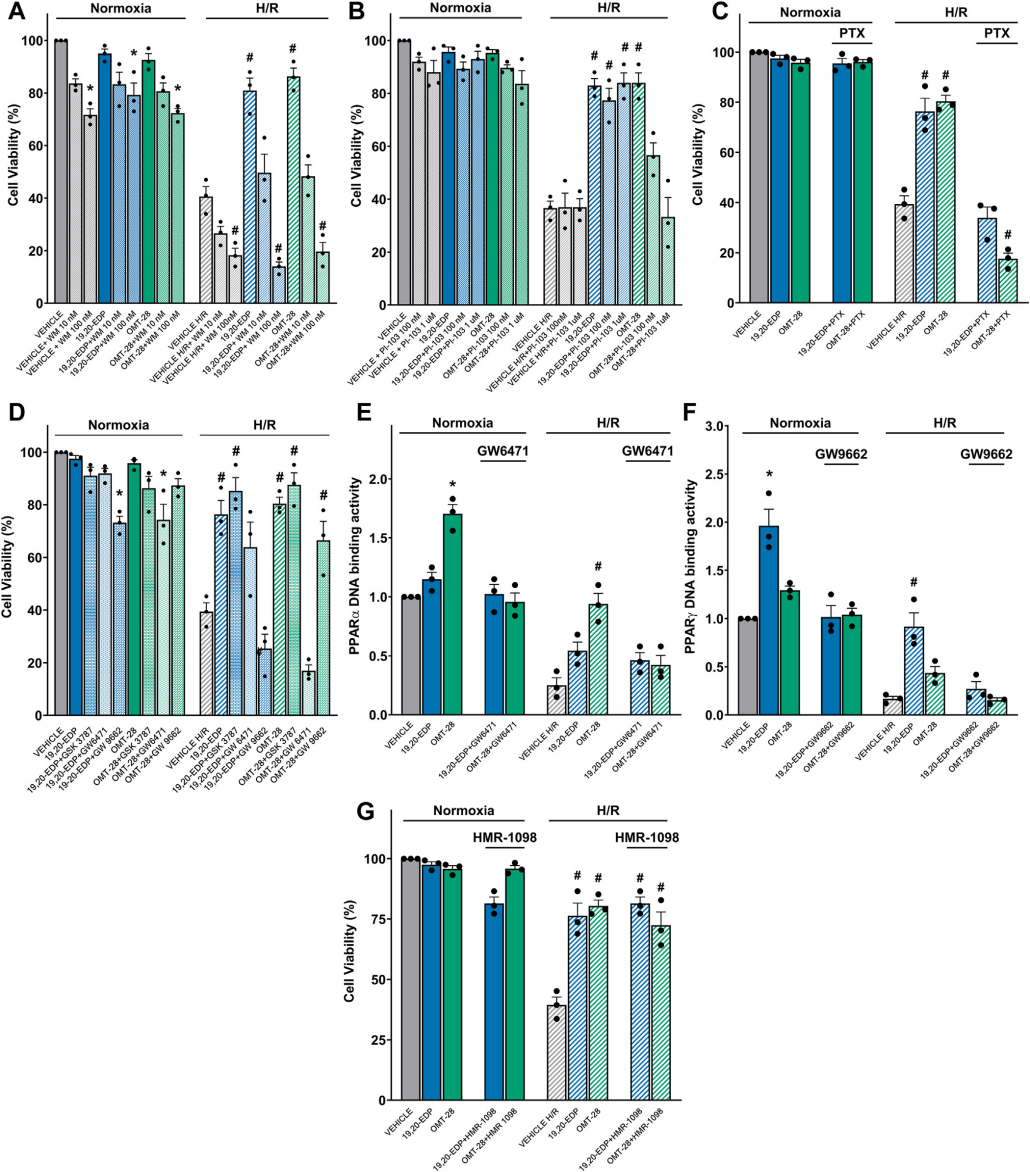
**OMT-28 protects NRCMs against HR injury through pleiotropic mechanisms.** The cytoprotective effect of OMT-28 was reduced by various inhibitors following HR injury in neonatal rat cardiomyocytes. NRCM subjected to normoxia or hypoxia-reoxygenation were treated with vehicle, 19,20-EDP (1 μM), or OMT-28 (1 μM) with or without inhibitors. *A*, pan-PI3K inhibition with Wortmannin (WM, 100 nM). *B*, PI3Kα-selective inhibition with PI-103 (100 nM). *C*, Gα_i_ inhibition with pertussis toxin (PTX, 200 ng/ml). *D*, PPAR inhibition with GSK3787 (1 μM), GW6471 (1 μM), or GW9662 (1 μM) to selectively inhibit isoforms β/δ, α, and γ, respectively. *E*, ELISA quantification of OMT-28-induced PPARα DNA-binding. *F*, ELISA quantification of 19,20-EDP-induced PPARγ DNA-binding. *G*, sKATP inhibition with HMR-1098 (10 μM). Values represent mean ± SEM, data were obtained by analyzing responses of three independent cell preparations and using at least three technical replicas, *p* < 0.05 statistically significant, one-way ANOVA, Bonferroni *post hoc* test, ∗ *versus* vehicle control normoxia; # *versus* vehicle control HR.

We first assessed the role of PI3K using wortmannin (WM), a broad PI3K class I, II, and III inhibitor (28, 29, 30). 100 nM WM pre-treatment almost completely ablated 19,20-EDP- and OMT-28-mediated cytoprotection against HR (from 2.2- to 1.2-fold, and 2.1- to 1.3-fold HR control viability, respectively) (Fig. 2*A*). However, this was confounded by the observation that WM pre-treatment alone was significantly cytotoxic at normoxia (0.7-fold normoxic control) and HR (0.5-fold HR control). To ascertain the role of PI3K more specifically, we utilized a PI3Kα-selective inhibitor, PI-103 (Fig. 2*B*) (31, 32). PI-103 pre-treatment alone was not significantly cytotoxic at any concentration compared to normoxic or HR controls. Interestingly, while PI-103 pre-treatment did not affect 19,20-EDP-mediated cytoprotection at any concentration, OMT-28-mediated cytoprotection was ablated by PI-103 in a concentration-dependent manner, suggesting that cytoprotection by OMT-28 but not 19,20-EDP is dependent on PI3Kα.

PTX was used to assess the involvement of Gα_i_ protein signaling in OMT-28-mediated protection against HR injury (33). PTX completely blocked both 19,20-EDP and OMT-28-mediated cytoprotection against HR injury (Fig. 2*C*). Additionally, OMT-28 in combination with PTX significantly worsened HR-induced loss of cell viability (0.5-fold HR control), suggesting additional cytotoxic mechanisms during HR injury that are not activated by PTX plus 19,20-EDP in HR injury nor PTX plus OMT-28 at normoxia.

PPARs are a family of nuclear hormone receptor transcription factors that have been shown to be targets of EpFA signaling (9, 25, 26, 34). To investigate the involvement of PPARs in our model, we used co-treatments with either GSK3787, GW6471, or GW9662 to selectively inhibit PPARs isoforms β/δ (35), α (36), and γ (37), respectively. Under normoxic conditions, non-significant reductions in cell viability were observed for both GSK3787 and GW9662 in combination with OMT-28, but OMT-28 plus GW6471 significantly reduced cell viability at normoxia (0.7-fold normoxic control) (Fig. 2*D*). Conversely, co-treatment of 19,20-EDP with GW9662 but not GSK3787 or GW6471 was cytotoxic at normoxia. These observations of cytotoxicity at normoxia likely do not fully account for the total blockage of GW6471 against OMT-28 mediated cytoprotection, as well as GW9662 completely blocking 19,20-EDP-mediated cytoprotection in HR injury. Interestingly, GW9662 only moderately blunted OMT-28-mediated cytoprotection against HR while GSK3787 had no effect. In contrast, 19,20-EDP-mediated cytoprotection was mildly inhibited by GW6471, while GSK3787 had no effect. This data suggests that OMT-28-mediated cytoprotection in HR injury is mostly dependent upon PPARα activity, while 19,20-EDP is dependent upon PPARγ activity. Supporting these observations, PPARα DNA binding activity was significantly increased by OMT-28 alone at normoxic conditions (1.7-fold normoxic control) but not 19,20-EDP, and this was completely blocked by GW6471 (Fig. 2*E*). OMT-28 also rescued PPARα DNA binding activity following HR injury (2.0-fold HR control) with this effect also being blocked by GW6471. Additionally, while 19,20-EDP had no effect on PPARα DNA binding activity, 19,20-EDP but not OMT-28 enhanced PPARγ DNA binding activity at both normoxia and HR, and this was completely blocked by co-treatment with GW9662 (Fig. 2*F*).

sK_ATP_ channels are known to be activated by arachidonic acid-derived epoxyeicosatrienoic acids (38, 39, 40). Interestingly, co-treatment with the selective sK_ATP_ channel inhibitor, HMR 1098 (41) did neither block 19,20-EDP- nor OMT-28-mediated rescue of cell viability against HR injury (Fig. 2*G*), demonstrating that OMT-28 and 19,20-EDP may utilize distinct signaling mechanisms compared to other cardioprotective EpFAs in NRCMs.

**Figure 3:**
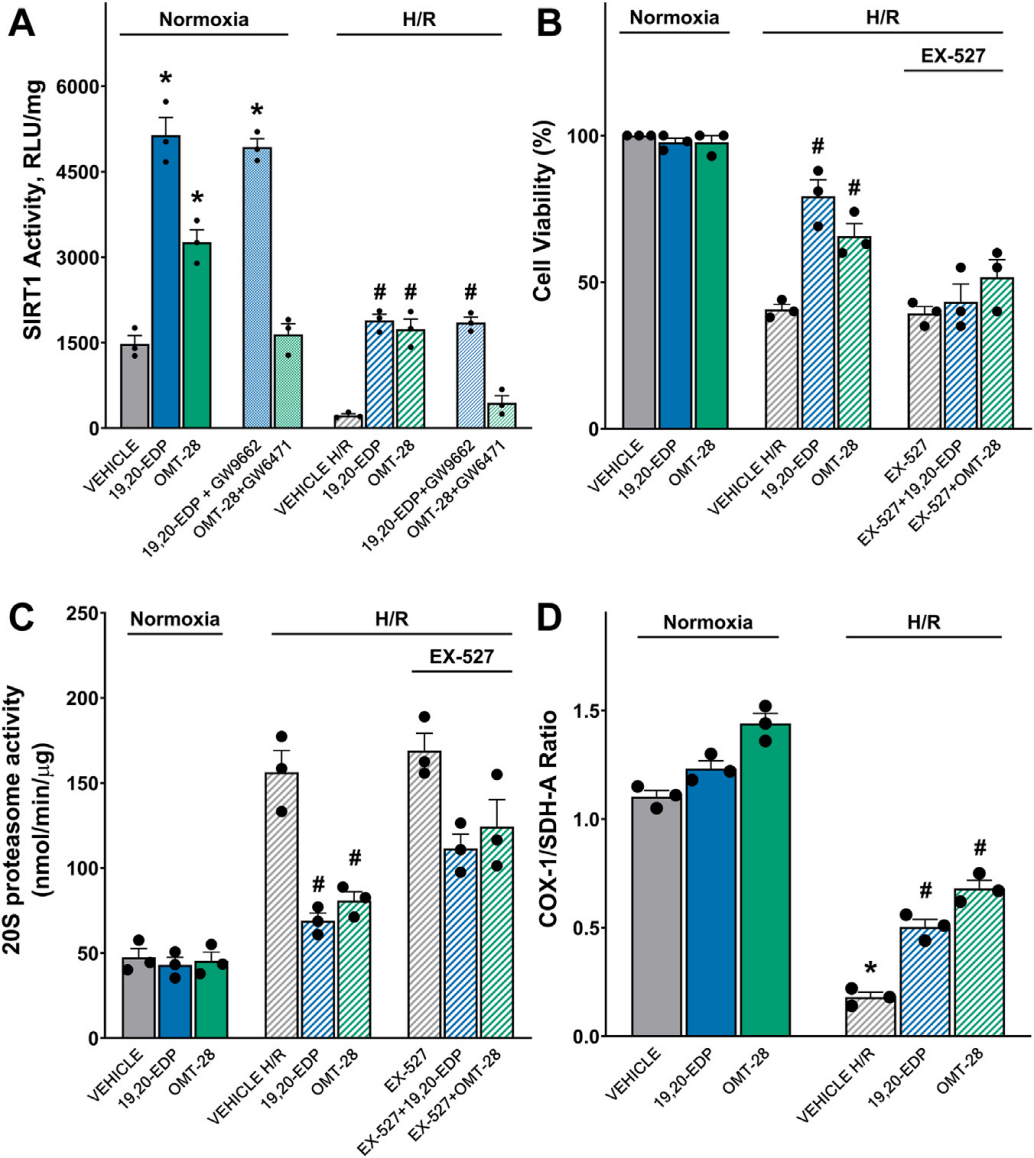
**Cytoprotection induced by OMT-28 is dependent upon PPARα-mediated preservation of SIRT1 activity.***A*, SIRT1 activity in NRCMs subjected to normoxia or HR injury and incubated with vehicle, 19,20-EDP (1 μM), OMT-28 (1 μM) with and without PPAR-inhibitors: GW9662 (PPARγ antagonist, 1 μM) and GW6471 (PPARα antagonist, 1 μM). HL-1 cells subjected to normoxia or HR injury and incubated with vehicle, 19,20-EDP (1 μM), OMT-28 (1 μM) or SIRT1 inhibitor (EX-527, 10 μM) were assessed for, *B*, cell viability in HL-1 cells, *C*, 20S proteasome activity in HL-1 cells. *D*, ELISA quantification of the SDH-A/COX-1 ratio as a marker of mitobiogenesis in NRCMs. Values represent mean ± SEM, data were obtained by analyzing responses of three independent cell preparations and using at least three technical replicas, *p* < 0.05 statistically significant, one-way ANOVA, Bonferroni *post hoc* test, ∗ *versus* vehicle control normoxia; # *versus* vehicle control HR.

SIRT1 activity has been associated with EpFA/PUFA signaling in several experimental models (17, 42, 43, 44). To assess the role of SIRT1 in OMT-28-mediated cardioprotection, we measured SIRT1 activity in NRCMs following normoxia or HR (Fig. 3*A*). Both 19,20-EDP and OMT-28 significantly increased SIRT1 activity under normoxia. While HR injury greatly ablated SIRT1 activity compared to normoxic control, both 19,20-EDP and OMT-28 rescued SIRT1 activity following HR. Interestingly, OMT-28-mediated SIRT1 activation was fully blocked by GW6471 in both normoxic and hypoxic conditions, demonstrating that OMT-28-mediated SIRT1 activation is dependent on PPARα activation (Fig. 3*A*). Conversely, 19,20-EDP-mediated SIRT1 activation was not blunted to any degree by GW9662 at either normoxia or HR, suggesting that 19,20-EDP-mediated SIRT1 activation is not dependent on PPARγ activation (Fig. 3*A*). In HL-1 cells, we demonstrated that the SIRT1-specific inhibitor EX-527 ablated OMT-28-mediated rescue of cell viability and 20S proteasome activity (Fig. 3, *B* and *C*). Finally, to assess the effect of OMT-28 on mitochondrial biogenesis, a process coupled to SIRT1 and PGC-1ɑ, we measured the ratio of protein levels of mitochondrial DNA-coded cytochrome C oxidase subunit 1 (COX-1) and nuclear DNA-encoded succinate dehydrogenase subunit A (SDH-A) in NRCMs (Fig. 3*D*) (45, 46). As expected, HR injury induced a pronounced drop in COX-1/SDH-A ratio (∼0.2-fold normoxic control). OMT-28 significantly rescued COX-1/SDH-A ratio following HR injury, and this was more potent compared to 19,20-EDP (3.8- and 2.5-fold HR control, respectively). In addition, only OMT-28 significantly increased COX-1/SDH-A ratio at normoxic conditions, while 19,20-EDP had no effect.

Overall, OMT-28-mediated cytoprotection against HR injury is dependent upon PI3Kα, Gα_i_, PPARα, and SIRT1 signaling pathways, with OMT-28-mediated SIRT1 activation being dependent upon PPARα activity. Activation of SIRT1 appears to have a meaningful impact on mitobiogenesis following HR injury. This data demonstrates clear differences in signaling mechanisms between OMT-28 and the endogenous comparator 19,20-EDP, despite both exhibiting comparable degrees of cytoprotection in cardiomyocyte HR injury models.

#### OMT-28 preserves mitochondrial function following cardiomyocyte HR injury

**Figure 4:**
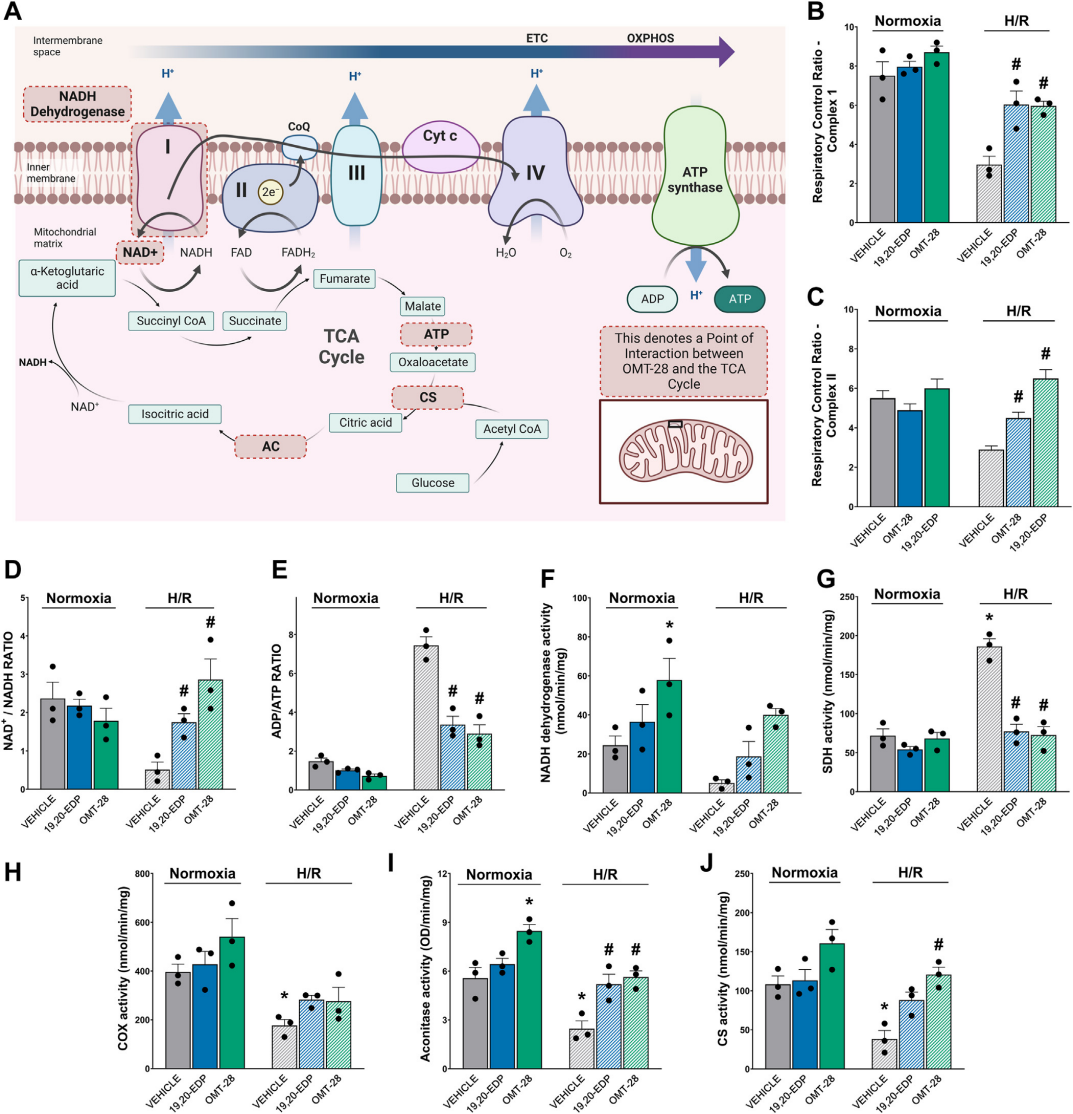
**OMT-28 improves mitochondrial function, ADP/ATP-, and NAD+/NADH-ratios.** Mitochondrial function was assessed in NRCMs subjected to normoxia or HR injury and incubated with vehicle, 19,20-EDP (1 μM), or OMT-28 (1 μM). *A*, schematic representing potential points of interaction between OMT-28, the TCA cycle and mitochondrial respiratory chain. *B*, complex I respiratory control ratio (RCR). *C*, complex II RCR. *D*, NAD+/NADH-ratios. *E*, ADP/ATP- ratio. *F*, NADH dehydrogenase activity. *G*, SDH enzymatic activity. *H*, COX-1 enzymatic activity. *I*, aconitase enzymatic activity. *J*, citrate synthase enzymatic activity. Values represent mean ± SEM, data were obtained by analyzing responses of three independent cell preparations and using at least three technical replicas, *p* < 0.05 statistically significant, one-way ANOVA, Bonferroni *post hoc* test, ∗ *versus* vehicle control normoxia; # *versus* vehicle control HR.

Among other EpFAs, 17,18-EEQ and 19,20-EDP have been shown to preserve mitochondria as part of their protective mechanisms of action (12, 14, 47). Thus, we hypothesized that OMT-28 also protects against HR-induced cardiac mitochondrial damage (Fig. 4*A*). Using permeabilized NRCMs following normoxia or HR injury, we first assessed the effect of OMT-28 on respiratory control ratio (RCR), a metric for mitochondrial efficiency (*i.e.*, coupling of ADP phosphorylation to respiration) by measuring the ratio between respiration rate when mitochondria are maximally converting ADP to ATP and respiration rate at minimal conversion of ADP to ATP (*i.e.*, respiration largely due to electron leak and non-mitochondrial oxygen consumption) (48). Complex I-mediated respiration experiments demonstrated that HR injury significantly reduced RCR from ∼7 to ∼2 in vehicle-treated NRCMs. Supporting our hypothesis, RCR was significantly rescued by either 19,20-EDP or OMT-28 (2.5-fold HR control) (Fig. 4*B*). We also observed a non-significant increase of complex I-mediated RCR to ∼8.5 by OMT-28 alone in normoxic conditions. Additionally, HR injury significantly reduced RCR from 5.5 to ∼3 in complex II-mediated respiration experiments in vehicle-treated NRCMs (Fig. 4*C*). Interestingly, only OMT-28 significantly rescued complex II-mediated RCR following HR injury (2.2-fold HR control), while 19,20-EDP-mediated restoration of RCR did not reach statistical significance (1.5-fold HR control). Additionally, no effect on complex II-mediated RCR was observed by 19,20-EDP or OMT-28 treatment at normoxic conditions. Supporting these observations, the HR-induced reduction in NAD+/NADH ratio (Fig. 4*D*) and the HR-induced increase in ADP/ATP (Fig. 4*E*) were both significantly blocked by either 19,20-EDP or OMT-28. However, OMT-28 appeared to improve ADP/ATP ratio in HR injury moderately better than 19,20-EDP (0.3-fold and 0.5-fold HR control, respectively).

To further investigate the mechanism of OMT-28-mediated preservation of mitochondrial function against HR injury, we assessed maximal activities of individual electron transport chain (ETC) and tricarboxylic acid cycle (TCA) enzymes in mitochondrial fractions from NRCMs. For ETC enzyme activity experiments, non-significant increases in complex I activity by 19,20-EDP or OMT-28 treatment were observed at both normoxic conditions and HR injury compared to their vehicle controls, although OMT-28 was more potent than 19,20-EDP in this manner (Fig. 4*F*). Additionally, the HR-induced elevation in complex II activity was significantly ablated by 19,20-EDP or OMT-28 (both 0.4-fold HR control), while no effects were observed at normoxic conditions (Fig. 4*G*). 19,20-EDP and OMT-28 only moderately restored complex IV activity following HR injury (both 1.6-fold HR control), but these both did not reach statistical significance (Fig. 4*H*). Aconitase activity was rescued to a similar degree by 19,20-EDP or OMT-28 following HR-injury (2.1- and 2.2-fold HR control, respectively), while only OMT-28 significantly increased aconitase activity (1.5-fold) at normoxic conditions (Fig. 4*I*). For TCA cycle enzyme activity experiments, both 19,20-EDP and OMT-28 appeared to rescue citrate synthase activity following HR injury in NRCMs (2.3-fold and 3.2-fold HR control, respectively), but only OMT-28 reached statistical significance and was also more potent than 19,20-EDP in this manner. OMT-28 also non-significantly increased citrate synthase activity at normoxic conditions (1.5-fold normoxic control) (Fig. 4*J*). Overall, OMT-28 provides equal or greater improvement of mitochondrial ETC or TCA cycle enzymatic activities compared to 19,20-EDP in HR injury.

#### OMT-28 pre- and post-treatment inhibits HR-induced mitochondrial ROS production

**Figure 5:**
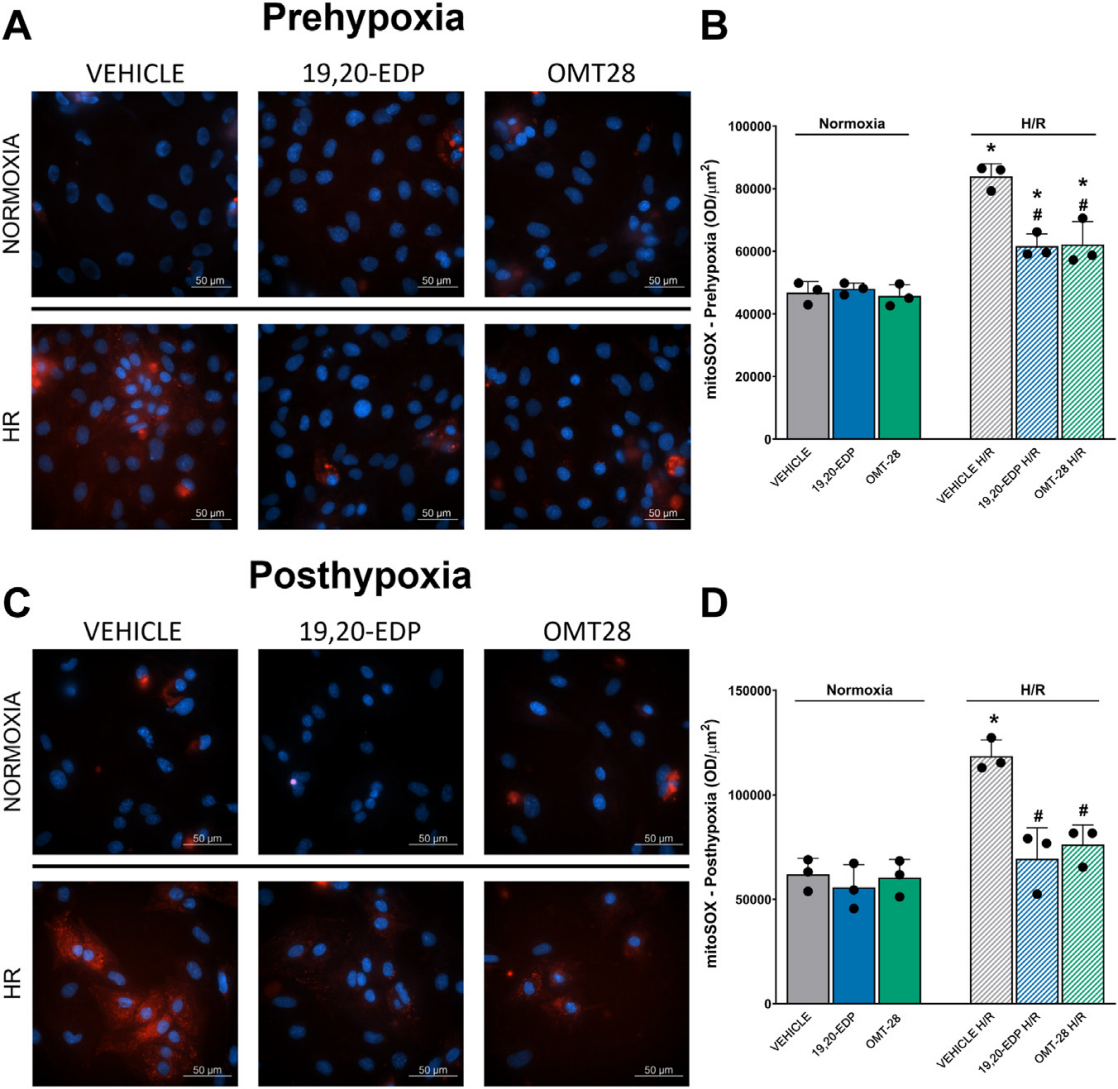
**OMT-28 limits HR-induced mitochondrial ROS production in NRCMs.** Representative images and quantification of mitochondrial ROS produced in NRCMs treated with vehicle, 19,20-EDP (1 μM), or OMT-28 (1 μM) before hypoxia or at the beginning of reoxygenation. *A*, pre-hypoxia treatment representative images. *B*, pre-hypoxia treatment ROS quantification. *C*, post-hypoxia treatment representative images. *D*, post-hypoxia treatment ROS quantification. Values represent mean ± SEM, data were obtained by analyzing responses of three independent cell preparations and using at least three technical replicas, *p* < 0.05 statistically significant, one-way ANOVA, Bonferroni *post hoc* test, ∗ *versus* vehicle control normoxia; # *versus* vehicle control HR. Image created with Biorender.com and published with permission.

Core to the pathogenesis of ischemic/hypoxic heart disease, accumulation of mitochondrial reactive oxygen species (mitoROS) and dysfunctional mitochondria during HR injury occur due to respiratory chain disruption and impaired autophagy (49, 50, 51, 52). Thus, we hypothesized that OMT-28 would suppress HR-induced mitoROS formation. Furthermore, we treated NRCMs with OMT-28 (or 19,20-EDP) at either the beginning of hypoxia (Fig. 5, *A* and *B*) or at re-oxygenation (Fig. 5, *C* and *D*) to assess the dependence of protection by OMT-28 on the phase of HR injury OMT-28 is added. In normoxia or HR-treated NRCMs stained with the mitochondrial superoxide-reactive dye MitoSOX, both 19,20-EDP and OMT-28 significantly blocked HR-induced MitoSOX fluorescence when treated at the beginning of hypoxia (0.7-fold HR vehicle control); however, MitoSOX fluorescence in all HR-treated groups remained significantly above levels observed in normoxic control. Interestingly, significantly lower MitoSOX fluorescence compared to HR control was also observed when either 19,20-EDP or OMT-28 were added at re-oxygenation (0.6-fold HR control). Although this was only moderately better protection compared to when drugs were added at the beginning of hypoxia, MitoSOX levels were also not significantly elevated compared to normoxic controls. Overall, these data suggest that OMT-28 can prevent HR-induced mitoROS formation when added either at the beginning of hypoxia or at re-oxygenation.

### OMT-28 protects cardiomyocytes against inflammatory injury

**Figure 6:**
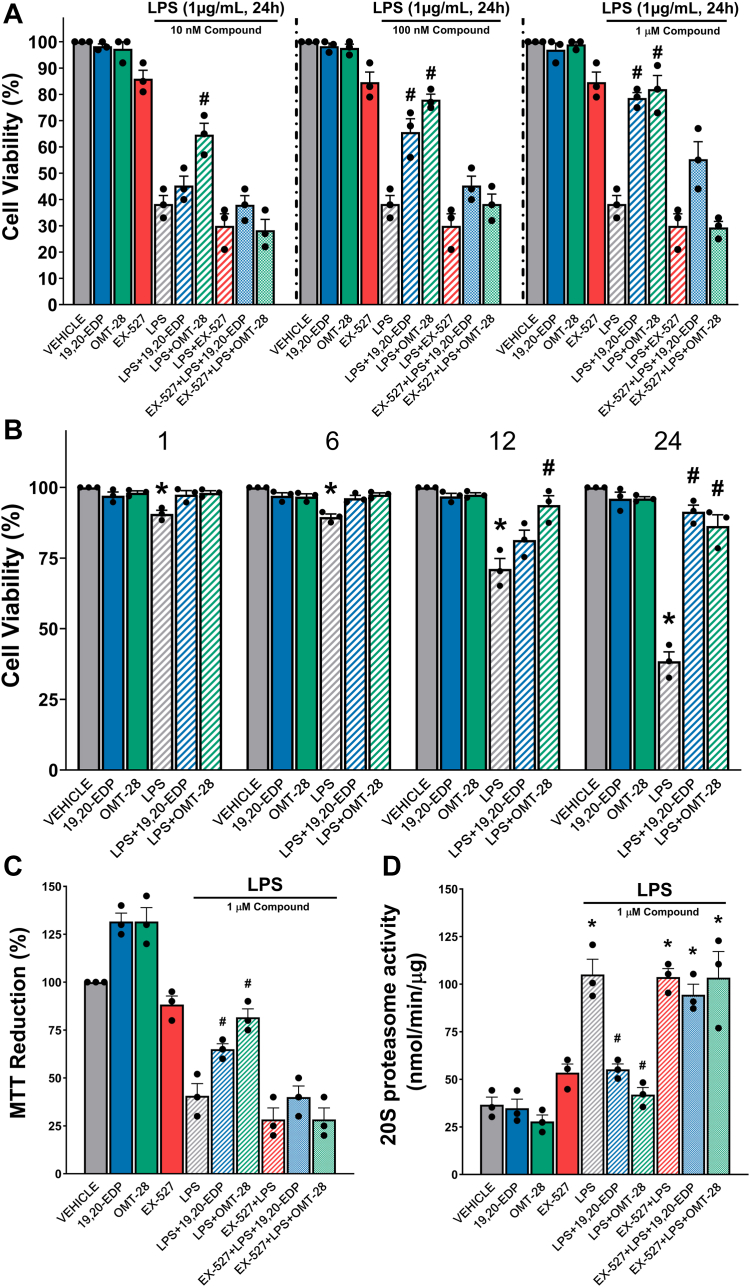
**OMT-28 limits LPS-induced cell death and injury in HL-1 cells.** Assessment of the cytoprotective effect of OMT-28 following LPS injury. *A*, cell viability (CCK-8) was assessed in cells exposed to 24 h LPS (1 μg/ml) and treated with either vehicle, 19,20-EDP or OMT-28 (10 nM, 100 nM, and 1 μM) with or without a SIRT1 inhibitor (EX-527,1 μM). *B*, cell viability (CCK-8) was assessed in cells exposed for 1, 6, 12 or 24 h LPS (1 μg/ml) and treated with either vehicle, 19,20-EDP or OMT-28 (1 μM). *C*, cell proliferation (MTT), and *D*, 20S proteasome activity in cells exposed to 24 h LPS (1 μg/ml) and treated with either vehicle, 19,20-EDP (1 μM) or OMT-28 (1 μM). Values represent mean ± SEM, data were obtained by analyzing responses of three independent cell preparations and using at least three technical replicas, *p* < 0.05 statistically significant, one-way ANOVA, Bonferroni *post hoc* test, ∗ *versus* control conditions; # *versus* LPS control.

Mitochondrial dysfunction and mitoROS production largely influence pro-inflammatory signaling, the degree of which greatly influences the severity and pathogenesis of myocardial ischemia-reperfusion injury (49, 53). We also speculated that OMT-28 would be cytoprotective and anti-inflammatory in an LPS-induced inflammatory injury model in HL-1 cells and NRCMs. After 24 h of LPS treatment, viability of HL-1 cells was reduced to ∼40% in the vehicle group. OMT-28 treated HL-1 cells showed a concentration-dependent response (60% (10 nM), 80% (100 nM) and 90% (1 μM), Fig. 6*A*). OMT-28 was cytoprotective against LPS challenge at all tested treatment times (1, 6, 12, and 24-h) in HL-1 cells, significantly rescuing cell viability (Fig. 6*B*). Both OMT-28 and control compound 19,20-EDP limited cell death as observed not only in CCK-8 viability experiments but also in MTT activation and 20S proteasome activity (Fig. 6, *C* and *D*). Both OMT-28 and 19,20-EDP increased overall mitochondrial oxidative capacity reflected by increased MTT reduction already under non-stressed conditions (Fig. 6). The observed cell protective effects, especially of OMT-28, were SIRT1 dependent, *i.e.*, Ex-527 blocked the OMT-28 mediated beneficial effects on cell viability, MTT reduction, and proteasome activity (Fig. 6). Interestingly, OMT-28 appeared to be more potent than 19,20-EDP in this manner, as OMT-28 began to significantly block LPS-induced loss of cell viability earlier (12 h) than 19,20-EDP (24 h).

**Figure 7:**
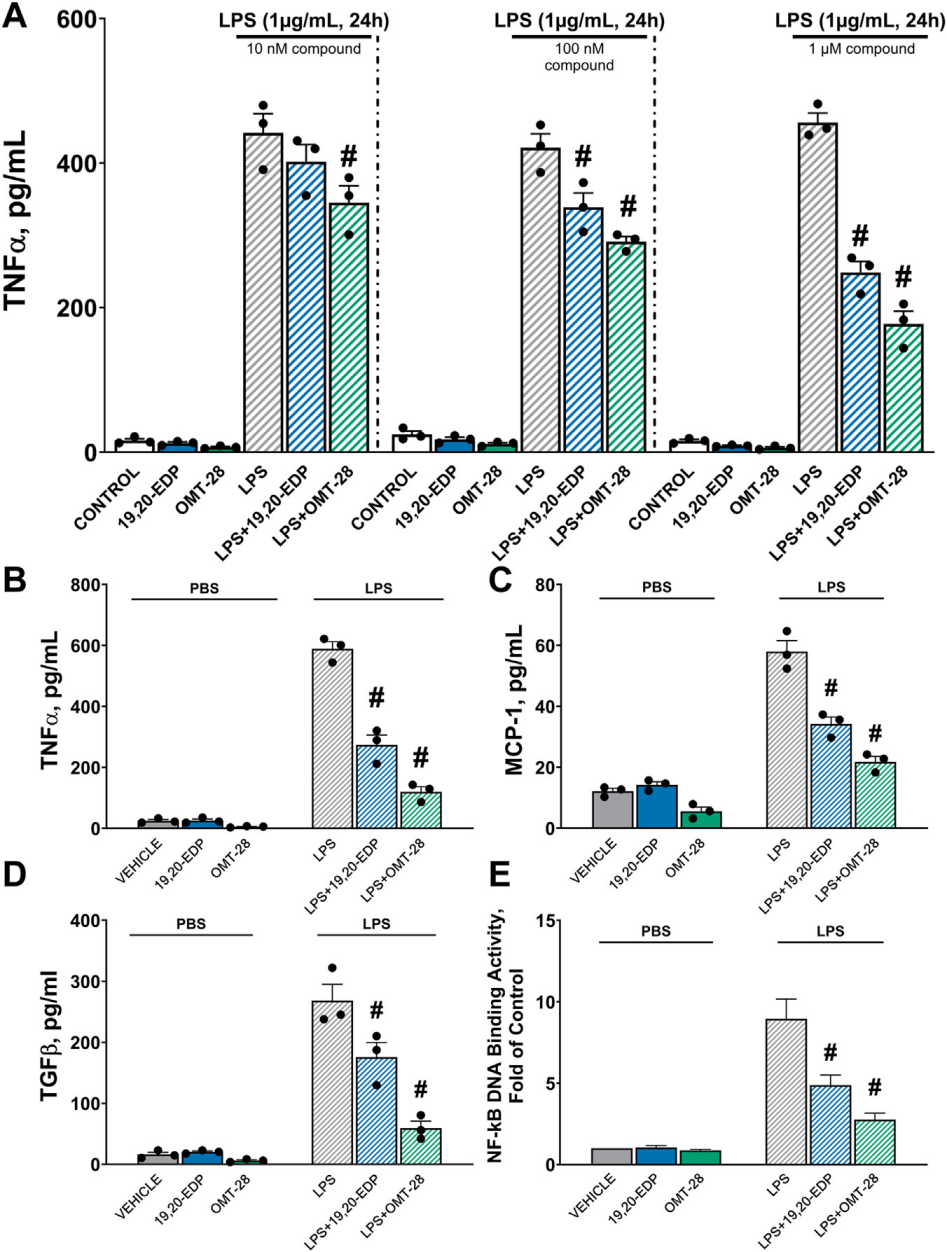
**OMT-28 limits LPS-induced inflammation in cardiac cells.** Analysis of several inflammatory markers expressed by cardiac cells exposed to 24 h LPS (1 μg/ml) and treated with either vehicle, 19,20-EDP or OMT-28. *A*, ELISA quantification of TNFα expression in HL-1 treated with either vehicle, 19,20-EDP or OMT-28 (10 nM, 100 nM, and 1 μM) following LPS insult. Analysis of inflammatory markers in NRCM. *B*, TNFα expression, *C*, MCP-1 expression, *D*, TGF-β expression, and *E*, NF-κβ DNA-binding activity. Values represent mean ± SEM, data were obtained by analyzing responses of three independent cell preparations and using at least three technical replicas, *p* < 0.05 statistically significant, one-way ANOVA, Bonferroni *post hoc* test, ∗ *versus* vehicle control in PBS treated cells: # *versus* vehicle control in LPS-treated cells.

To investigate the anti-inflammatory potential of OMT-28, we measured the levels of several cytokines in HL-1 and NRCM cell culture media following 24-h LPS challenge. Similar to trends observed in the cell viability data, both 19,20-EDP and OMT-28 significantly blocked LPS-induced secretion of TNFα (Fig. 7, *A* and *B*), MCP-1 (Fig. 7*C*), and TGF-β (Fig. 7*D*), but OMT-28 was more potent in all cases when compared to 19,20-EDP. Lastly, we investigated whether these cytoprotective and anti-inflammatory effects of OMT-28 could involve differences in the activation of NF-κB, a transcription factor that mediates transcription of inflammation-related genes (54, 55). We measured NF-κB DNA binding activity in NRCMs during the acute inflammatory phase (at 1 h) of LPS-challenge and observed that both 19,20-EDP and OMT-28 significantly blocked LPS-induced NF-κB DNA binding activity (Fig. 7*E*). OMT-28 was more potent than 19,20-EDP here as well, supporting our cell viability and cytokine secretion data.

Overall, these data suggest OMT-28 possesses greater cytoprotective and anti-inflammatory efficacy than 19,20-EDP in HL-1 cells and NRCMs challenged with LPS. Furthermore, OMT-28 demonstrates protective properties in multiple *in vitro* cardiac injury models, potentially mediated through shared signaling pathways (*e.g.*, mitochondrial damage, innate inflammation, ROS generation).

### OMT-28 improves post-ischemic contractile recovery and inhibits inflammasome activation in isolated murine hearts

#### OMT-28 improves post-ischemic contractile recovery

As OMT-28 exhibited marked cardioprotection in multiple *in vitro* cardiac injury models, we tested the functional relevance of these data using ischemia-reperfusion (IR) injury-subjected isolated murine hearts perfused in the Langendorff-mode. Additionally, we looked for indications of a cell death pathway commonly associated with IR injury, the NLRP3 inflammasome (56), a multi-protein complex that mediates pyroptotic cell death *via* proteolytic caspase-1 activation, and subsequent maturation of the pro-inflammatory cytokine interleukin-1ꞵ (57). Thus, NLRP3 inflammasome inhibition has become a novel therapeutic target in myocardial IR-injury, with promising results emerging from preclinical studies and clinical trials (58, 59, 60). Considering the cytoprotective and anti-inflammatory effects of OMT-28 *in vitro*, we also hypothesized that OMT-28 may be cardioprotective in myocardial IR injury through inhibition of the NLRP3 inflammasome. In this regard, we compared the cardioprotective effects of OMT-28 in isolated murine hearts with that of the specific NLRP3 inflammasome inhibitor MCC-950, which has already been demonstrated to be cardioprotective in several IR injury models (61, 62).

**Figure 8:**
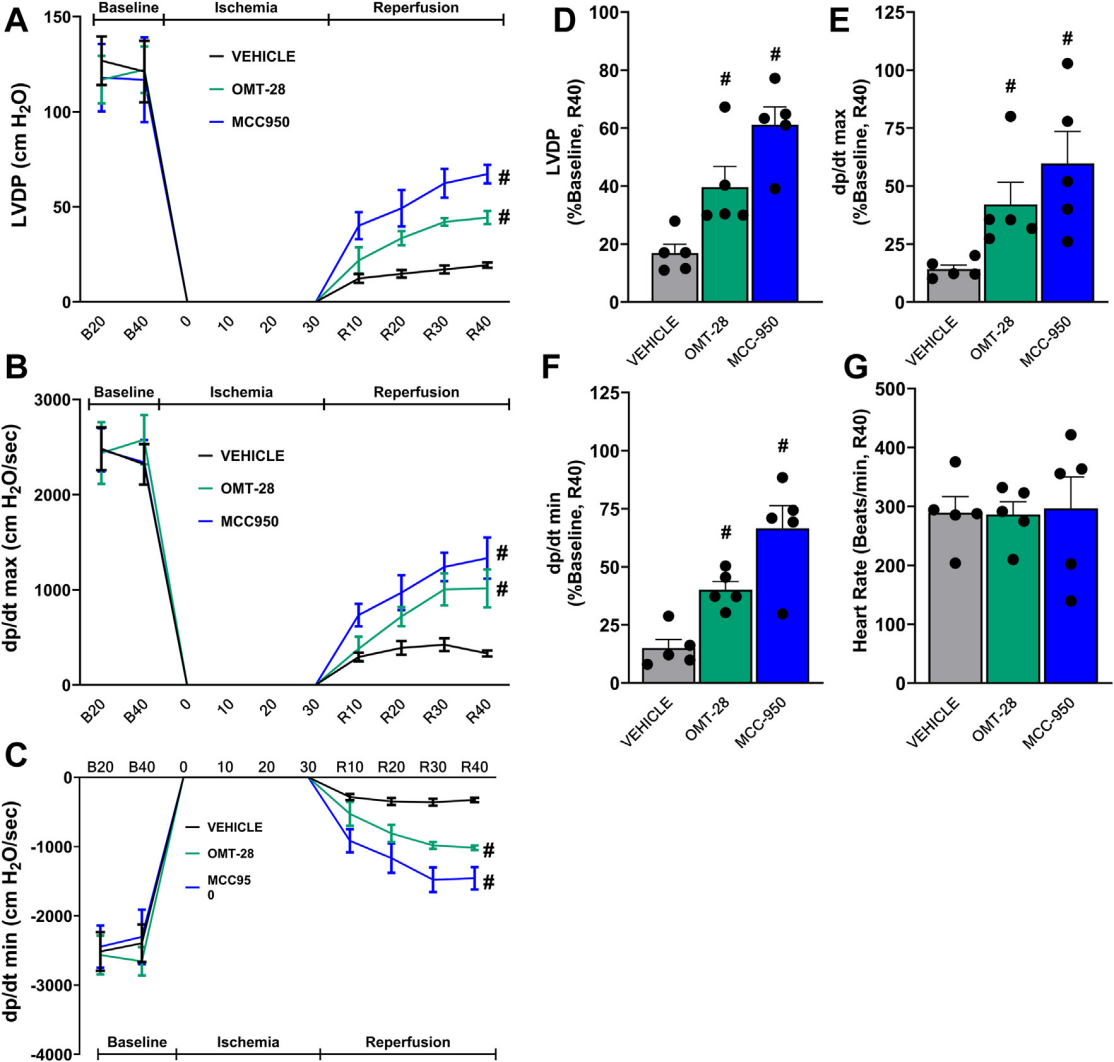
**OMT-28 enhances postischemic-reperfusion myocardial function recovery.** Perfusion of hearts with OMT-28 (1 μM) or MCC950 (1 μM) resulted in improved postischemic functional recovery. *A*, left ventricular developed pressure (LVDP) at baseline before drug treatment (B_20_), during ischemia, and at 10, 20, 30, and 40 min following reperfusion (R_10_, R_20_, R_30_, and R_40_). *B*, rate of contraction (dP/dt max). *C*, rate of relaxation (dP/dt min). *D*, LVDP recovery at 40 min reperfusion as a percentage of baseline. *E*, rate of contraction at 40 min reperfusion as a percentage of baseline. *F*, rate of relaxation at 40 min reperfusion as a percentage of baseline. *G*, heart rate assessed as beats per minute (BPM) at the end of reperfusion (R_40_). Values represent mean ± SEM, n = 6, *p* < 0.05 statistically significant, one-way ANOVA, Bonferroni *post hoc* test, # *versus* IR vehicle control.

Supporting our *in vitro* data, both OMT-28 and MCC-950 significantly rescued myocardial functional parameters when compared to IR control hearts. OMT-28-treated hearts had a percent left ventricular developed pressure (%LVDP of baseline) 2-fold higher than IR controls, 40 min after reperfusion (R40), while MCC950-treated hearts had 3-fold higher %LVDP at R40 compared to IR controls (Fig. 8, *A* and *D*). Similarly, both IR-induced reduction of the rate of contraction (dp/dt max) (Fig. 8, *B* and *E*) and rate of relaxation (dp/dt min) (Fig. 8, *C* and *F*) at R40 were significantly rescued by either OMT-28 or MCC-950, while heart rates at R40 were not significantly different between any treatment group (Fig. 8*G*). Overall, OMT-28 rescued post-ischemic myocardial function to a similar degree compared to NLRP3 inflammasome inhibition by MCC-950. This data demonstrates the functional relevance of our *in vitro* observations, providing important evidence that OMT-28 is cardioprotective against IR injury at both the cellular and organ level.

#### OMT-28 prevents IR-induced mitophagic responses associated with mitochondrial damage

Mitophagy is a housekeeping mechanism through which damaged mitochondria are cleared *via* recruitment to autophagosomes followed by subsequent lysosomal fusion and degradation, allowing the recycling of macromolecules for mitobiogenesis and preventing the accumulation of inflammogenic material (63). Thus, activation of mitophagy is a well-known response to mitochondrial damage in myocardial IR injury (64, 65). As OMT-28 strongly protected mitochondria against HR injury *in vitro*, we assessed mitochondrial fractions from isolated murine hearts for expression of mitophagic markers following IR injury.

**Figure 9:**
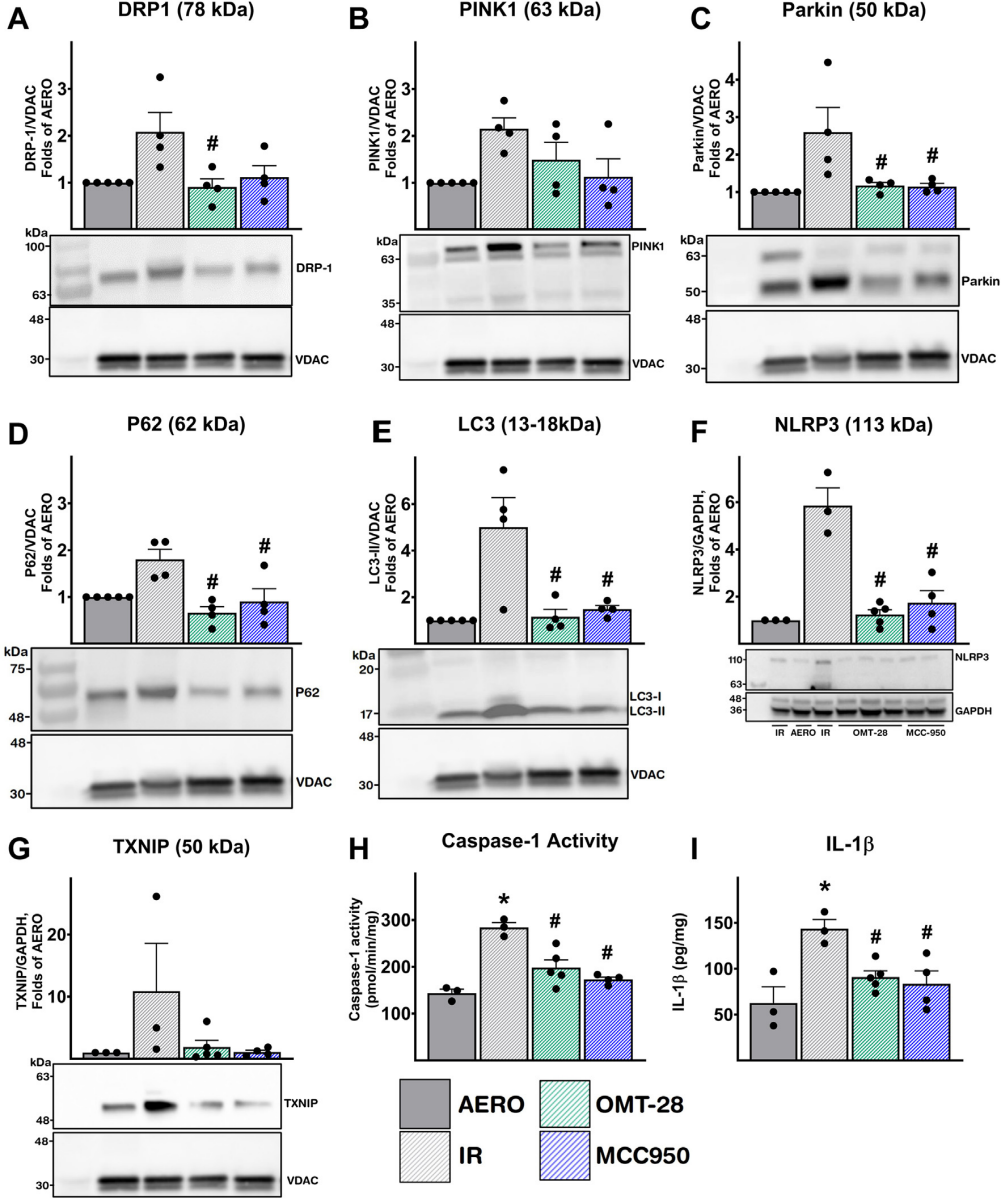
**OMT-28 limits IR-induced inflammasome and autophagy responses.** Perfusion of hearts with OMT-28 (1 μM) or MCC950 (1 μM) inhibited IR-induced activation of mitochondrial autophagy and the NLRP3 inflammasome. Representative immunoblots and densiometric quantification of mitochondrial autophagy-associated markers and NLRP3 inflammasome were assessed by immunoblotting. Protein expression was normalized to either VDAC (mitochondria) or GAPDH (cytosolic) loading controls. IR-induced mitochondrial expression of *A*, dynamin-related protein-1 (DRP-1), *B*, PTEN-induced kinase 1 (PINK1), *C*, Parkin, *D*, p62 and, *E*, microtubule-associated proteins 1A/1B light chain 3B (LC3B-II). Immunoblots were re-probed for multiple markers. The representative images for *A*, *B*, and *G* share the same tissue source. The representative images for *C*, *D*, and *E* share the same tissue source. IR-induced cytosolic expression of *F*, Nucleotide NLR family pyrin domain containing 3 (NLRP3) and *G*, thioredoxin interacting protein (TXNIP). *H*, cardiac caspase-1 enzymatic activity assessed in the cytosolic fraction following IR injury. *I*, cardiac quantification of interleukin-1β levels (IL-1B) expression following IR injury. Values represent mean ± SEM, n = 3 to 6, *p* < 0.05 statistically significant, one-way ANOVA, Bonferroni *post hoc* test, ∗ *versus* aerobic control (AERO); # *versus* IR vehicle control.

Levels of dynamin-related protein-1 (DRP1), a protein involved in mitochondrial fission, were markedly upregulated in mitochondrial fractions from IR control hearts (2-fold higher than aerobic control) (Fig. 9*A*). This is expected as DRP1 accumulation in mitochondria followed by fission is an early mechanism to separate damaged mitochondria for mitophagy (66, 67). Supporting our previous observations, mitochondrial DRP1 in OMT-28-treated hearts was significantly lower than IR control hearts, being restored to levels comparable to aerobic controls. Mitochondrial DRP1 in MCC950-treated hearts was also restored to near-aerobic control levels, although this was not significantly lower compared to IR control-treated hearts.

PINK1 accumulates on mitochondrial outer membranes because of mitochondrial damage, leading to recruitment and subsequent activation of Parkin, an E3 ubiquitin ligase. Parkin ubiquitinates mitochondrial surface proteins, promoting direct binding of autophagy receptors such as p62 (68, 69). A significant 2.1-fold increase in mitochondrial PINK1 levels was observed in IR control hearts compared to aerobic controls (Fig. 9*B*). Mitochondrial PINK1 levels in OMT-28 and MCC950-treated hearts were not significantly higher than aerobic controls (1.4- and 1.1-fold higher, respectively), although these were also not significantly different when compared to IR control hearts. Lastly, mitochondrial Parkin levels were 2.6-fold higher in IR control hearts compared to aerobic controls, but OMT-28 or MCC950 significantly blocked IR-induced Parkin accumulation in mitochondrial fractions (Fig. 9*C*).

Following the detection of mitochondrial damage, ubiquitination of selective proteins on the surfaces of mitochondria mediates specific targeting of these mitochondria to mitophagy. The autophagy receptor protein, p62, acts as an adaptor between these ubiquitinated proteins and the lipidated protein LC3-II, recruiting these mitochondria to mature autophagosomes and subsequent degradation (68, 70). We observed that both p62 (Fig. 9*D*) and LC3-II (Fig. 9*E*) were heavily accumulated in mitochondrial fractions from IR control hearts (1.8- and 5-fold higher than aerobic controls, respectively), suggesting IR-induced activation of mitophagy. Both OMT-28 and MCC950 treatment significantly decreased both mitochondrial p62 and LC3-II compared to IR controls.

Overall, these data show that improved myocardial post-ischemic functional recovery by treatment with OMT-28 is associated with a reduction of IR-induced mitophagy markers, suggesting that OMT-28 limited mitochondrial damage also in this *ex vivo* model. Specifically, PINK1/Parkin-dependent mitophagy and classical recruitment of p62 and LC3-II to mitochondria appear to be upregulated following IR injury and ameliorated with the addition of OMT-28.

#### OMT-28 prevents IR-induced accumulation of NLRP3 inflammasome activation markers

Past works suggest that the NLRP3 inflammasome is a target of EpFA compounds as a cardioprotective mechanism in myocardial IR injury (12). Thus, we assessed cytosolic fractions from isolated murine hearts for markers of NLRP3 inflammasome activation. As previously observed in our model, cytosolic NLRP3 levels were strongly upregulated by IR injury (6-fold aerobic control). Strikingly, OMT-28 or MCC-950 restored cytosolic NLRP3 to levels almost equivalent to aerobic controls, and these were significantly lower when compared to IR control hearts (Fig. 9*F*).

We also assessed cytosolic levels of thioredoxin-interacting protein (TXNIP). TXNIP and thioredoxin form an inhibitory complex at basal conditions, but as IR-induced oxidative stress ablates the thioredoxin-TXNIP interaction, TXNIP becomes elevated in the cytosol and directly interacts with NLRP3 to promote inflammasome activation (71). We observed an 11-fold increase in cytosolic TXNIP levels in IR control hearts compared to aerobic control, although high variance within the IR control group prevented these groups from being significantly different (Fig. 9*G*). However, OMT-28 or MCC-950 appeared to ablate IR-induced elevation of cytosolic TXNIP, restoring levels comparable to aerobic control hearts.

Following canonical NLRP3 inflammasome activation, caspase-1 is a key mediator of pyroptotic cell death and inflammatory cytokine maturation (72, 73, 74). Thus, we measured caspase-1 activity in cytosolic fractions from isolated murine hearts. Supporting our previous observations, caspase-1 activity levels were significantly elevated in IR control hearts (1.9-fold higher compared to aerobic control), while OMT-28 and MCC950 successfully blocked IR-induced caspase-1 activation (Fig. 9*H*). Furthermore, we measured cytosolic levels of IL-1β, a pro-inflammatory cytokine that is proteolytically processed in the cytosol by caspase-1 prior to its secretion. As expected, IR hearts exhibited 2.4-fold higher cytosolic IL-1β levels compared to aerobic controls, and this was significantly reduced by OMT-28 or MCC950 treatment (Fig. 9*I*).

Overall, these data demonstrate that OMT-28-mediated protection of isolated murine hearts against myocardial IR injury is associated with reduced NLRP3 inflammasome activation markers. These data support the cytoprotective and anti-inflammatory effects of OMT-28 observed *in vitro* and suggest that NLRP3 inflammasome inhibition could be an important mechanism involved in OMT-28-mediated cardioprotection.

## Discussion

**Figure 10:**
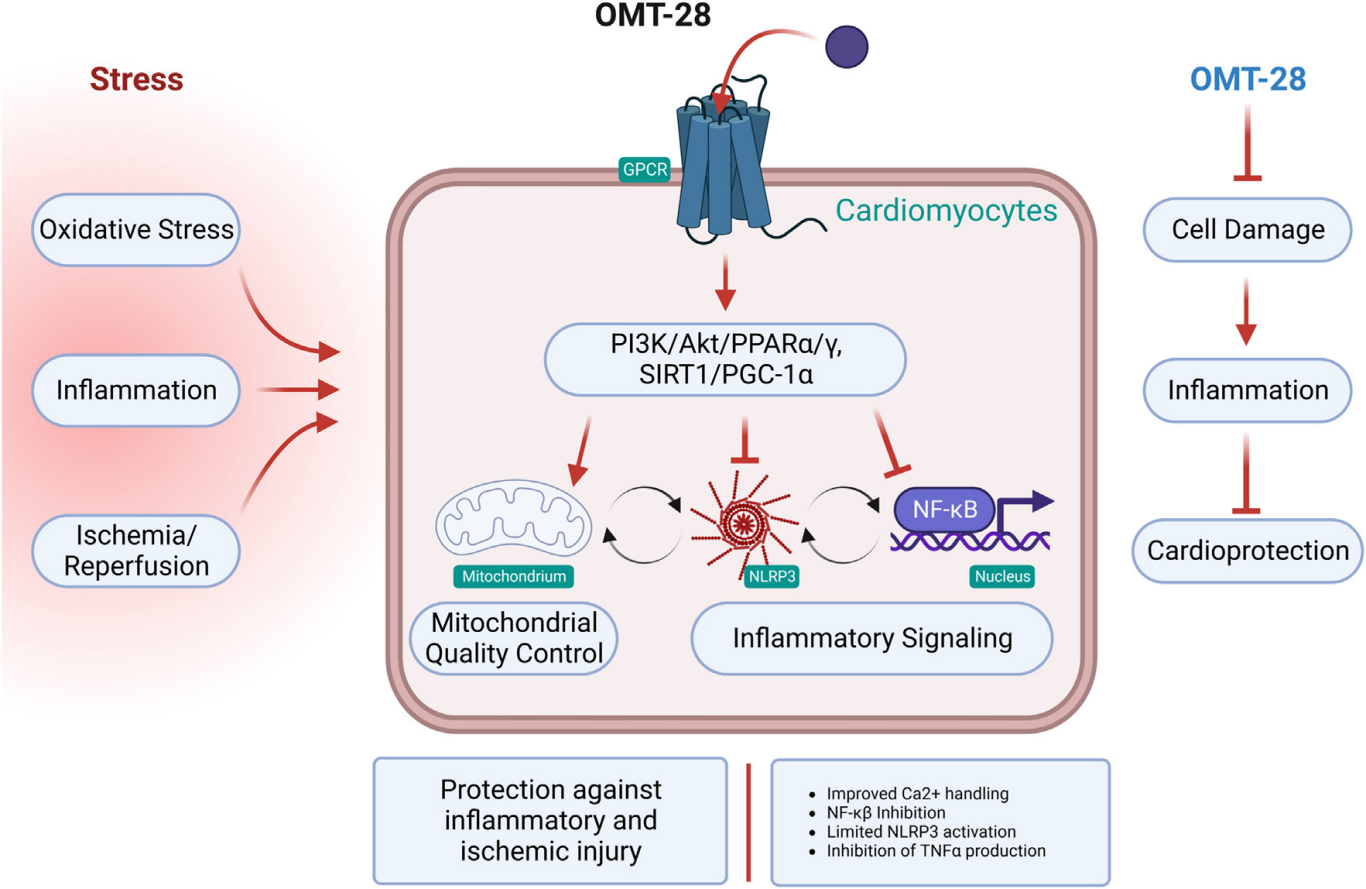
**Schematic of OMT-28 protective mechanisms.** Conceptual illustration demonstrating the potential cardioprotective role OMT-28 has toward inflammatory and ischemic injury. Hearts subjected to stressors such as excessive inflammation or ischemic injury have decreased viability, decrease mitochondrial quality, and elevated inflammatory responses leading to reduced function. OMT-28 protective effects are mediated *via* an unknown GPCR leading to activation of an intracellular signaling pathway involving PPARα and SIRT1 activation that preserves mitochondrial quality and limits inflammation resulting in a robust cardioprotective response. Image created with Biorender.com and published with permission.

This study shows that OMT-28, a synthetic analog of ω-3 epoxyeicosanoids, exhibits similar and, in part, even superior cytoprotective and anti-inflammatory properties compared to its natural counterparts. The beneficial effects of OMT-28 relied on its ability to ameliorate stress-induced mitochondrial dysfunction and inflammasome activation (Fig. 10), as revealed by our experiments in cultured cardiomyocytes and isolated perfused hearts.

OMT-28 was developed based on structure-activity-relationship (SAR) studies that measured the effect of synthetic 17,18-EEQ analogs on cardiomyocyte contraction as a marker of their biological activity (15, 16). The findings of the present study extend and support our previous hypothesis on the potential therapeutic use of OMT-28 in conditions of arrhythmia and cardiomyopathy (75). At the same time, these SAR studies revealed how the chemically and metabolically labile 17,18-EEQ can be rendered into a more drug-like compound by reducing the number of double bonds, replacing the epoxy group with an epoxy-bioisoster, and introducing a 3-oxa group (see Fig. 1*A* for a comparison of 17,18-EEQ and OMT-28 structures). These specific chemical modifications resulted in compounds with largely improved oral bioavailability, metabolic stability, and even slightly increased effects on cardiomyocyte contraction (16). Furthermore, in these studies, OMT-28 displayed efficacy in the low nanomolar range when tested in short-term experiments measuring its effects on the beating rate of NRCMs (15, 16). However, OMT-28 shows high protein binding (15, 16). Therefore, higher concentrations are required to achieve long-term effects in cardiomyocytes cultured in the presence of FBS. As shown in Figures 6 and 7*A*, OMT-28 exerts significant anti-inflammatory effects already at a concentration of 10 nM and this effect is saturated at a concentration of 1 μM. In contrast, 19,20-EDP starts to be effective only at a concentration of 100 nM. Importantly, the present study demonstrated for the first time that the same chemical modifications also maintain and improve the cardioprotective and anti-inflammatory properties of natural ω-3 epoxyeicosanoids. Similar strategies were successful in generating metabolically robust and functionally active analogs of other endogenous EpFAs (76, 77).

In our current study, OMT-28 and 19,20-EDP markedly ameliorated HR- and LPS-induced injury in both immortalized (HL-1) and primary cardiomyocytes (NRCM). At the molecular level, OMT-28 mediated effects in the HR model involved PI3Kɑ, Gɑ_i_, PPARɑ, and SIRT1. 19,20-EDP signaling was dependent on the same components but required PPARγ instead of PPARα. SIRT1 was also essential for the cytoprotective effect of OMT-28 in the LPS model, where OMT-28 reduced DNA binding of NF-κB and proinflammatory cytokine expression.

Our initial experiments revealed that the cardioprotective properties of 17,18-EEQ are stereospecific and become more pronounced in the presence of sEH–inhibitors (Fig. 1). In contrast, 19,20-EDP, the DHA-derived omega-3 epoxide, was sufficiently stable and effective in racemic form. Therefore, we decided to use 19,20-EDP instead of the parental 17(R),18(S)-EEQ as a comparator to OMT-28 throughout all further experiments. Comparing the biological activities of 19,20-EDP and OMT-28, we observed not only common features but also differences in their mode of action (MoA). Whereas common features included all global cytoprotective and anti-inflammatory effects, distinct differences became obvious regarding the signaling pathways activated by the DHA-derived natural epoxide and the synthetic analog of 17,18-EEQ (Table 1). OMT-28 mediated effects in the HR model involved PI3Kɑ, Gɑi, PPARɑ, and Sirt1. 19,20-EDP signaling was similarly blocked by inhibitors against Gɑi (PTX) and Sirt1 (Ex-527) but required PPARγ instead of PPARα and was not affected by the PI3K inhibitor PI-103. Extending the common features at the molecular level, Sirt1 was also essential for the cytoprotective effects of 19,20-EDP and OMT-28 in the LPS model, where both compounds reduced DNA binding of NF-κB and pro-inflammatory cytokine expression.

**Table 1:** Comparison of modes of action for 19,20-EDP and OMT-28

Global effect	OMT-28	19,20-EDP
Protection against HR-injury	+	+
Protection against LPS-induced endotoxemia	+	+
IR induced inflammasome activation	+	+ (12)
Mitochondrial function	++	+
Mitochondrial ROS production (mitoSOX)	−	−
Inflammatory cytokine production and NFkB activation	− −	−
Signaling component involved (inhibitor)		
PI3K inhibitor (PI-103)	Yes	No
G alpha i (PTX)	Yes	Yes
PPAR alpha (GW6471)	Yes	No
PPAR gamma (GW9662)	No	Yes
Sirt1 (EX-527)	Yes	Yes

“+” increased, “−“ decreased.

Our current mechanistic insight is primarily based on pharmacological inhibition experiments indicating whether certain components are essential for the MoA of OMT-28. However, it remains a major question for future studies whether OMT-28 and its natural counterparts directly or indirectly interact with the molecules mentioned above. In particular, identification of the primary target(s) of OMT-28, 19,20-EDP or 17,18-EEQ, remains elusive, similar to the limited reports for other EpFAs (78, 79, 80, 81). Evidence suggests that 17,18-EEQ could act through the prostacyclin receptor (IP) to sensitize neuronal TRPV1 and TRPA1 receptors (82). Another group proposed that ethanolamide derivatives of both 17,18-EEQ and 19,20-EDP suppress neuroinflammation *via* the endocannabinoid receptor 2 coupled to beta-arrestin activity (83). Other groups demonstrated a role for sK_ATP_ channels in ω-3 epoxyeicosanoid-mediated vasodilation (38, 84, 85); however, we did not observe any tangible effect of sK_ATP_ inhibition upon OMT-28’s ability to protect NRCMs. Further data from the current study indicates the protective action of OMT-28 was blocked by PTX treatment, suggesting the involvement of a G-protein coupled receptor that transduces signals to Gɑ_i_ proteins (86, 87). Interestingly, a previous study found that antagonizing PI3K with wortmannin abrogated the cardioprotective effects exerted by the PPARɑ agonist WY-14643 against IR injury in male Wistar rats (88). In line with this finding, we observed that OMT-28 mediated protection was attenuated following inhibition of PI3K.

Activation of SIRT1 may be key for understanding the mode of action of OMT-28. Supporting this hypothesis, we found that OMT-28 increased SIRT1 activity in cardiomyocytes already under normal conditions and limited its decline after HR and LPS treatment. Importantly, pharmacological SIRT1 inhibition blocked the cytoprotective and anti-inflammatory effects of OMT-28 as shown in both models. These findings are in line with other reports showing an important role of SIRT1 in activating adaptive responses toward stressors such as HR injury (42, 89, 90). SIRT1 may be also involved in mediating the effects of OMT-28 on mitochondrial biogenesis (91) and inhibition of pro-inflammatory transcription factor NF-κB (92, 93). The mechanism of how OMT-28 increases SIRT1 activity is unclear. Surprisingly, we found that GW6417 blocked this effect, suggesting the involvement of PPARα. As known from previous studies of other authors, enhanced SIRT1 expression is indeed inducible by fenofibrate, a classic PPARα activator (94). However, we cannot exclude that OMT-28 physically interacts with SIRT1 and that GW6417 acts as an antagonist at this level of potential short-term activation.

Important to sirtuin function is the ratio between NAD^+^/NADH, which can be increased or decreased by nutrient supply, energy consumption and hypoxia impacting mitochondrial function (90, 95, 96, 97, 98, 99, 100, 101). In general, OMT-28 was superior to 19,20-EDP in attenuating the loss of mitochondrial respiration stemming from HR injury as reflected in better RCR, NAD^+^/NADH, and ADP/ATP ratios. A similar trend was observed regarding electron transport chain enzymatic activities and TCA activity, following HR injury. Our recently published studies indicate that 19,20-EDP protects the heart from IR through attenuation of oxidative stress and protection of mitochondrial function (12, 18, 102). Consistently, both OMT-28 and 19,20-EDP attenuated mitochondrial ROS production following reoxygenation. Together, the current data supports a concept where OMT-28 enhances and sustains optimal mitochondrial quality following HR injury. Taking 19,20-EDP as an example, we speculate that OMT-28 activates not only SIRT1 but also SIRT3 which deacetylates MnSOD and thereby directly reduces O_2_^−^-production in mitochondria (13, 102). Rapid post-translational activation of MnSOD would also be in line with our finding that OMT-28 acutely inhibits O_2_^−^-production if added only in the reoxygenation phase.

Our current results also demonstrate that OMT-28 improved the post-ischemic functional recovery of isolated perfused mouse hearts subjected to IR injury in a Langendorff apparatus. This cardioprotective effect involved limiting NLRP3 inflammasome activation associated with caspase-1 activation and subsequent IL-1β expression, similarly, as reported before with 19,20-EDP (12, 13). NLRP3 inflammasome activation has been generally known to play a central role in myocardial IR injury (103). Indeed MCC950, a selective NLRP3 inflammasome inhibitor (104), was similarly effective as OMT-28 in our IR model. We used heart homogenates for analysis and thus do not know the type of cells in which OMT-28 inhibited inflammasome activation. Major candidates are cardiac fibroblasts that have been identified as the primary site of NLRP3 expression and function in IR injury of mouse hearts (105, 106). The mechanism of how OMT-28 inhibited IR-induced NLRP3 activation is likely related to the abilities of OMT-28 to reduce mitochondrial dysfunction and mitochondrial ROS production (107, 108, 109). Consistent with reduced mitochondrial damage, OMT-28 also prevented IR-induced mitophagic responses. Additionally, OMT-28 attenuated IR-induced up-regulation of PINK1, parkin, p62, and LC3-II, which are involved in the selective clearance of damaged mitochondria (60, 110).

In conclusion, our results demonstrate the ability of OMT-28 to prevent mitochondrial dysfunction and inflammasome activation, and thus to protect cultured cardiomyocytes and isolated hearts against HR-injury and inflammatory stress. Further preclinical and clinical studies are needed to prove the therapeutic relevance of our findings.

## Experimental procedures

### Animals

All studies were carried out using 2- to 3-month-old male C57/Bl6 mice weighing 25 to 30 g. Mice were maintained in a colony at the University of Alberta and housed under conditions of constant temperature and humidity with a 12:12-h light-dark cycle. Mice were fed on a standard rodent chow diet ad libitum (fat 11.3%, fiber 4.6%, protein 21% (w/w)). The composition of the diet includes linolenic acid (0.27%), linoleic acid (2.12%), arachidonic acid (0.01%), omega-3 fatty acid (0.45%), total SFA (0.78%), and total MSFA (0.96%) (PicoLabRodent Diet 20 Cat. No 5053, LabDiets, Inc). All animal experimental protocols were approved by the University of Alberta Health Sciences Welfare Committee (University of Alberta Animal Welfare, ACUC, study ID#AUP330) and conducted according to strict guidelines provided by the Guide to the Care and Use of Experimental Animals (Volume. 1, 2nd ed., 1993, from the Canadian Council on Animal Care).

### Preparation of test compounds and controls

OMT-28 was synthesized as described previously (see compound-4 in (15)) and provided for this study by OMEICOS (Batch No. MC1102T). 19,20-EDP (0468130-13) and 17,18-EEQ (0473839-7) were purchased from Cayman Chemicals (Ann Arbor, MI). The enantiomers of 17,18-EEQ were resolved and prepared using chiral-phase HPLC as described previously (1, 111). The compounds were prepared in 100% ethanol as 1 mM stock solutions and stored in a desiccator at −80 °C. Pertussis toxin (PTX) (Sigma, P6659) (112) and LPS (Sigma, Batch No. 036M4070V) were prepared using PBS (Gibco, 10010-023). HMR1098 (Sigma, A8292) (84) and wortmannin (WM) (Calbiochem, 681675) (113) were prepared in 100% ethanol. PI-103 (Cayman, 1008208) (113), GW9662 (Sigma, M6191) (114), GW6471 (Sigma, G5045) (115), GSK3787 (Sigma G7423) (116), t-AUCB (Cayman 16568) (18) and EX527 (Sigma, E7034) (44) were prepared in DMSO. Ethanol and DMSO solvents were used as vehicle controls for *in vitro* and *ex vivo* experiments respectively. Unless stated otherwise, OMT-28, epoxy-fatty acid (EpFA) compounds, HMR1098, GSK3787, GW6471, GW9662, and EX-527 were added at the start of HR injury experiments, while PTX was pre-treated for 2 h prior, WM and PI-103 were pre-treated for 3 h prior to HR injury experiments (Table 2).

**Table 2:** Inhibitors used to elucidate functional dependencies of OMT-28 and 19,20-EDP

Compound name	Target	Form of inhibition	Concentration(s)	Catalogue #	References for use in cardiac cells
EX-527	SIRT1	Selective	10 and 100 nM	Sigma E7034	(44)
GSK3787	PPARβ/δ	Selective, Irreversible	1 μM	Sigma G7423	(116)
GW6471	PPARα	Selective	1 μM	Sigma G5045	(115)
GW9662	PPARγ	Selective, Irreversible	1 μM	Sigma M6191	(114)
HMR-1098	sKATP	Selective	10 μM	Sigma A8292	(84)
PI-103	PI3Kα	Selective	100 nM and 1 μM	Cayman 1008208	(113)
Pertussis Toxin	Gα_i_	Selective	200 ng/ml	Sigma P6659	(112)
t-AUCB	sEH	Selective	1 μM	Cayman 16568	(116)
Wortmannin	PI3K	Non-selective	100 nM	Calbiochem 681675	(113)

### Cultivation of HL-1 cardiac cells

HL-1 cardiac cells were a kind gift from Dr Claycomb. Cells were maintained at 37 °C in a humidified atmosphere of 5% CO_2_ and 95% air as well as cultivated in Claycomb medium supplemented with 10% FBS (Sigma, F1051), norepinephrine (0.1 mM) (Calbiochem, 324900) and glutamate (2 mM). The cells were cultivated until they achieved about 80% confluency on plastic flasks coated with fibronectin. Cells with 80% confluency were used to perform experiments, all data using HL-1 cells were obtained by analyzing responses of three independent cell preparations and using at least three technical replicas.

### Isolation of neonatal rat cardiomyocytes (NRCM)

Rat neonatal cardiomyocytes were isolated and cultivated in DMEM supplemented with 15% horse serum, 5% fetal bovine serum, and 1% penicillin and streptomycin, as previously described (117).

### Hypoxia-reoxygenation (HR)

As described previously, HL-1 cardiac cells or NRCMs were exposed to either normoxia or 24 h hypoxia (1% O_2_/5% CO_2_) followed by 6 h reoxygenation (21% O_2_/5% CO_2_) using a digitally controlled atmospheric cell culture chamber (17).

### Lipopolysaccharide (LPS) model

Exposure to environmental stressors can cause mitochondrial dysfunction leading to irreversible cardiac damage. Exposure to broadly used concentrations of LPS has been well documented to be one of the major culprits to damage cardiac mitochondria and promote cardiac dysfunction. HL-1 cardiac cells or NRCMs were exposed to either vehicle or LPS (1 mg/ml) for 6 or 24 h. LPS was purchased from Sigma-Aldrich and dissolved in phosphate-buffered saline (PBS).

### Subcellular fractionation

Following experimental treatment, mouse hearts, HL-1 cells, and NRCMs were homogenized, and portioned into subcellular fractions. Briefly, samples were ground with a mortar and pestle on dry ice and then homogenized in an ice-cold homogenization buffer (20 mmol/l Tris-HCL, 50 mmol/l NaCl, 50 mmol/l NaF, 5 mmol/l sodium pyrophosphate, 1 mmol/l EDTA and 250 mmol/l sucrose were added on the day of the experiment, pH 7.0). Samples were first centrifuged at 800*g*, 4 °C for 10 min to separate cellular debris. The supernatant was collected and centrifuged further at 10,000*g*, 4 °C for 20 min. Mitochondrial-enriched fractions were obtained from the resuspension of the resulting pellet in the homogenization buffer. Further ultra-centrifugation of the supernatant at 105,000*g*, 4 °C for 60 min yielded the cytosolic fraction.

### Langendorff isolated hearts

Male C57BL/6 mice (2–3 months old) were anesthetized by an intraperitoneal injection of sodium pentobarbital (Euthanyl, 100 mg/kg). Following complete non-responsiveness to external stimulation, hearts were quickly excised and perfused in the Langendorff mode with Krebs-Henseleit buffer containing (in mM) 120 NaCl, 25 NaHCO_3_, 10 Dextrose, 1.75 CaCl_2_, 1.2 MgSO_4_, 1.2 KH_2_PO_4_, 4.7 KCL, 2 Sodium Pyruvate (pH 7.4) and bubbled with 95% O_2_ and 5% CO_2_ at 37 °C. The left atrium was then excised, and a water-filled balloon made of saran plastic wrap was inserted into the left ventricle through the mitral valve. The balloon was connected to a pressure transducer for continuous measurement of LVDP and heart rate (HR). Hearts with persistent arrhythmias were excluded from the experiment. Mouse hearts were perfused in the retrograde mode at a constant flow rate for 40 min of baseline (stabilization) and then subjected to 30 min of global no-flow ischemia followed by 40 min of reperfusion. Hearts were perfused with either vehicle (Krebs buffer with 0.1% DMSO), OMT-28 (1 μM), or MCC950 (1 μM; Sigma Aldrich, cat# PZ0280). In all experiments, chemicals were added 20 min before ischemia and were present in the heart throughout the reperfusion period. The percentage of left ventricular developed pressure (%LVDP) at 40 min of reperfusion (R40), as compared to baseline LVDP, was taken as a marker for recovery of contractile function. After 40 min of reperfusion, hearts were immediately frozen in liquid nitrogen and stored at −80 °C. Hemodynamic parameters were acquired and analyzed using ADI software.

### Cellular analysis

#### Cell viability

Cell viability was evaluated using a commercially available cell counting kit-8 (CCK-8) (Millipore-Sigma, 96992) assay. Briefly, cellular dehydrogenases on plasma membranes of live cells can oxidize WST-8 (2-(2-methoxy-4-nitrophenyl)-3-(4-nitrophenyl)-5-(2,4-disulfophenyl)-2H-tetrazolium, monosodium salt), producing a water-soluble formazan dye which upon reduction in the presence of an electron carrier, 1-Methoxy PMS, creates formazan. The amount of formazan, measured spectrophotometrically at 590 nm, positively correlates with the number of viable cells.

An MTT assay was employed to examine mitochondrial oxidative metabolism as previously described in the assay kit (Abcam, CT02). The intensity of reduction of 3-(4,5-dimethylthiazol-2-yl)-2,5-diphenyltetrazolium bromide to formazan crystals by mitochondrial dehydrogenases positively correlates with overall oxidative metabolic activity (118). The optical density of DMSO-extracted formazan was measured spectrophotometrically at 595 nm.

#### Proteasome activity

Total 20S proteasome activity assay was determined in cytosolic fractions monitoring the release of AMC by proteolytic cleavage of the peptide Suc-LLVY-AMC (APT280, Chemicon) by 20S proteasomes. The kit measures the formation of 7-Amino-4-methylcoumarin (AMC) from the cleavage of substrate LLVY-AMC by the proteasome. Fluorescence was monitored at wavelengths of 380 nm (excitation) and 460 nm (emission). Specific activities were determined from a standard curve established with AMC peptide.

#### Aconitase

Aconitase enzyme activity was measured in tissue lysates spectrophotometrically (Abcam, ab109712). Aconitase converts aconitate to cis-aconitate, which can be detected by increases in absorbance (240 nm) in the tissue homogenates. The amount of cis-aconitate formed is proportional to aconitase activity.

#### Reactive oxygen species (ROS)

ROS production was assessed using a commercially available bioluminescent assay (Promega Corp, ROS-Glo H_2_O_2_ Assay, G8820) according to the manufacturer’s instructions. Briefly, cells underwent experimental treatments in a 96-well plate. After treatment cells were incubated with an assay detection solution containing a derivatized luciferin substrate for 20 min and then relative luminescence was assessed. H_2_O_2_ was added as a positive control.

#### Sirtuin-1 activity assay

SIRT1 enzymatic activity was measured in cells following experiments using a SIRT-Glo assay kit (Promega Corp, G6470). Briefly, cells underwent experimental treatments in a 96-well plate. After treatment cells were incubated with an assay detection solution containing an acetylated luminogenic peptide substrate to determine deacetylase activity. The assay was modified by incubation for 20 min with the SIRT1-specific inhibitor EX-527 (10 μM) before initiation of the reaction. SIRT1-specific activity was determined by assessing the difference in deacetylase activity with and without the inhibitor.

#### Caspase-1 activity assay

Cleavage of the caspase-1 specific fluorogenic substrate Ac-YVAD-AMC (Enzo life Sciences, ALX-260-024-M005) was used to assess functional caspase-1 activity in cytosolic fractions of the heart homogenates. Briefly, the cytosolic fractions (20–30 μg protein) were incubated with the fluorogenic substrate in a reaction buffer (50 mM HEPES, 100 mM NaCl, 0.5% CHAPS, 1 mM EDTA, 10% glycerol, 10 mM DTT) and the fluorescence intensity of the cleaved 7-Amino-4-methylcoumarin (AMC) was quantitated kinetically over 1 h at 37 °C using a fluorometer (excitation 380 nm, emission 460 nm wavelengths). The activity was calculated by using a linear standard curve created with AMC and normalized to the sample protein concentration.

#### Cytokine and DNA binding activity assays

Enzyme-linked immunosorbent assay (ELISA) was used to quantify the cardiac cytosolic levels of the cytokine IL-1ꞵ (Abcam, ab100705) according to the manufacturer’s instructions. Briefly, cytosolic samples (30–40 μg protein) were pipetted into a 96-well plate and incubated for 2.5 h at room temperature where IL-1ꞵ present in a sample became attached to the wells by the immobilized antibody specific for mouse IL-1ꞵ that is coated on the wells. The wells were then washed and a biotinylated anti-mouse IL-1ꞵ antibody was added and incubated for 1 h at room temperature with gentle shaking. Horseradish peroxidase (HRP) conjugated streptavidin was added to the wells after washing away unbound biotinylated antibodies and incubated for 45 min at room temperature with gentle shaking. A 3,3′,5,5′-tetramethylbenzidine (TMB) substrate solution was then added to the wells and incubated for 30 min at room temperature in the dark with gentle shaking. Afterwards, the stop solution was pipetted into the wells and the intensity of the color was measured at 450 nm. IL-1ꞵ concentration in the different samples was calculated by using a linear standard curve created with different concentrations of the standard IL-1ꞵ.

Cell culture medium was centrifuged for 5 min at 5000*g*, and supernatants were analyzed by ELISA for TNFa (Abcam, ab100747) and MCP-1 levels (Abcam, ab208979). Briefly, samples were added into individual wells of a 96-well plate coated with a TNFa mouse-specific antibody. After washing, wells were incubated with HRP-conjugated streptavidin, washed, and incubated with substrate solution. The intensity of the color was measured spectrophotometrically at 450 nm. Increased color intensity occurred in a linear proportion to the amount of TNFa or MCP-1 in the samples. Transforming growth factor-beta 1 (TGF-β1) was measured in the cell culture supernatants by ELISA kit (Abcam, ab119558).

NF-kB DNA binding assays were performed using an ELISA kit (Active Motif), in which nuclear extracts from treated cells were assessed for DNA binding activity. Briefly, cells were gently harvested after 1 h LPS challenge with PBS and centrifuged for 2 min at 500*g*, the supernatant was removed, and the remaining cell pellets were washed with PBS. The cell pellet was resuspended in 200 μl of buffer (75 mM NaCl, 1 mM NaH2PO4, 8 mM Na2HPO4, 250 mM sucrose, 200 μg/ml digitonin, including protease inhibitors 0.5 mM PMSF, 2 μg/ml leupeptin, 0.2 pg/ml aprotinin and 2.5 μg/ml pepstatin). 10% Nonidet P-40 (0.5% final) was added to cell suspensions, vortexed for 10 s, and centrifuged for 30 s at 30*g*. Pellets were incubated on ice for 30 min on a rocker and subsequently centrifuged at 14,000*g* for 10 min at 4 °C. The supernatant was used as a nuclear extract.

PPARγ (ab133101) and PPARɑ (ab133107) DNA binding activity were measured in nuclear extracts obtained from cells following experiments using ELISA kits (Abcam). Briefly, the assays were based on the specific recognition of affixed peroxisome proliferator response elements (PPREs) by PPARγ or PPARɑ transcription factors contained in cell lysates. Relative levels of binding can then be assessed by the addition of specific primary antibodies directed against bound PPAR proteins. Colorimetric readouts at 450 nm were obtained following the addition of a secondary antibody conjugated to HRP.

#### Mitochondrial function

To test overall mitochondrial oxidative metabolism, we measured the ADP/ATP ratio in cell lysates using a luciferase-based method (Sigma-Aldrich, MAK135). Briefly, ADP and ATP are released from cells following lysis with the kit’s working reagent. Luciferase and D-luciferin immediately begin reacting with ATP to produce light. The intensity of the light corresponds with ATP concentration. Secondly, cellular ADP is later enzymatically converted to ATP. A second intensity of light can be detected through the same mechanism. The ratio of these two light intensities represents the ratio of total ADP and ATP within the sample.

NAD/NADH ratio in cell lysates was assessed using a bioluminescent kit (Promega, G9071). Briefly, samples are first lysed and then treated with an NADH-dependent reductase enzyme that reduces proluciferin to luciferin. Luciferin is then quantified by the addition of the Ultra-Glo Recombinant Luciferase as the produced light signal corresponds to the concentration of luciferin.

Mitobiogenesis was evaluated using an ELISA kit (Abcam, ab110217) based on simultaneous detection of succinate dehydrogenase (SDH-A), a subunit of Complex II (nuclear DNA-encoded protein) and cytochrome c oxidase subunit 1 (COX-1), a subunit of Complex IV (mitochondrial DNA-encoded protein). The ratio between these proteins (COX-1/SDH-A) reflects the intensity of mitobiogenesis.

Mitochondrial respiration was measured in saponin-permeabilized HL-1 cells or NRCM using a Clark oxygen electrode connected to Oxygraph Plus recorder (Hansatech Instruments Ltd). Briefly, following experimental treatment (HR protocol) cells were first washed with ice-cold PBS, then gently trypsinized (0.25%) for 5 min, collected, centrifuged to remove trypsin (300*g* for 1 min), resuspended in respiration buffer (20 mM HEPES, 10 mM KH_2_PO_4_, 0.5 mM EGTA, 3 mM MgCl_2_.6H_2_O, 20 mM taurine, 1 g/l BSA, 60 mM potassium-lactobionate, 110 mM mannitol, 0.3 mM dithiothreitol, pH 7.1 adjusted with 5 N KOH) and kept on ice until adding to oxygraph chamber. Cells were permeabilized directly in the oxygraph chamber by adding saponin to a final concentration of 10 μg/ml. This allows rapid diffusion of test compounds or inhibitors in the cells. To measure specific respiration, respiratory substrates were added to the chamber at different times. Respiration rates were measured at 30 °C before and after the addition of 2 mM ADP in the presence of 5 mM malate and 10 mM glutamate as respiratory substrates, normalized to protein concentrations. Respiratory control ratio (RCR) was calculated as the ratio between basal and ADP-stimulated respiration rates.

*Complex I*: Malate (5 mM) and glutamate (5 mM). The substrates were prepared as pH neutral, aliquoted, and frozen. The airtight chamber was closed, and respiration was recorded with a Clark electrode. After 5 to 10 min of baseline recording, malate and glutamate were added directly into the injection port on top of an airtight chamber using a syringe. Next, another 5 to 10 min of recording was obtained and then ADP (2 mM) was added to stimulate ADP-dependent respiration. The kinetic oxygen consumption was recorded for another 5 to 10 min. *Complex II*: Succinate was prepared in neutral pH buffer, aliquoted, and frozen. The final concentration in the chamber was 10 mM. The procedure of oxygen consumption measurement was identical to that described for Complex I.

### Immunoblotting

Immunoblot analysis was conducted as previously described (119). Briefly, following the resolution of proteins *via* electrophoresis with (10–15%) SDS-polyacrylamide gels, they were transferred to polyvinylidene difluoride (PVDF) membranes (BioRad Laboratories, 1620177) which were then blocked with 5% non-fat milk in TBS-T buffer (0.15 M NaCl, 3 mM KCl, 25 mM tris hydroxymethyl methylamine and 0.1% tween-25, pH 7.4). Membranes were washed three times with TBS-T buffer and then incubated overnight at 4 °C with primary antibodies. Following secondary washing with TBS-T and incubation with horseradish-peroxidase linked anti-rabbit IgG secondary antibodies, membranes were visualized with chemiluminescence substrates. Immunoblots were probed with antibodies against LC3B (1:1000, Cell Signaling Technology, Inc, cat# 3868), Parkin (1:500, Cell Signaling Technology, Inc, cat# 2132), DRP1 (1:500, Cell Signaling Technology, Inc, cat# 5391), NLRP3 (1:500, Cell Signaling Technology, Inc, cat# 15101) P62 (1:1000, Abcam, cat# ab56416), PINK1 (1:500, Abcam, cat# ab23707), TXNIP (1:500, MBL International Co, cat# K0205-3), VDAC (1:2000, Abcam, cat# ab14734), AMPKɑ (1:1000, Cell Signaling Technology, Inc, cat# 25332), p-AMPKɑ, Thr172 (1:1000, Cell Signaling Technology, Inc, cat# 2535), SDH-A (1:1000, Cell Signaling Technology, Inc, cat# 5839), COX IV (1:5000, Cell Signaling Technology, Inc, cat# 11967), p-AKT (1:1000, Cell Signaling Technology, Inc, cat# 4060), GSK (1:1000, Cell Signaling Technology, Inc, cat# 9315), β-Actin (1:2000, Cell Signaling Technology, Inc, cat# 4967), and GAPDH (1:2000, Cell Signaling Technology, Inc, cat# 2118, 5174). Relative band intensity to control was measured using Image J software.

### Microscopy

Mitochondrial ROS production was assessed in NRCM after being subjected to 24 h hypoxia. Briefly, NRCM were cultured on glass-bottom 35-mm dishes pre-coated with laminin (1 mg/ml in PBS) for 12 h (suitable for fluorescent microscopy (MatTek Corp, 6-well glass bottom)) at 60 to 70% confluency and treated with indicated reagents. NRCM were treated with vehicle (culture media with 0.1% Ethanol), OMT-28 (1 μM), or 19,20-EDP(1 μM) either before hypoxia or at the beginning of reoxygenation. The dishes were placed in a micro incubator (37 °C, 21% O_2_/5% CO_2_) installed on the objective stage of the microscope. Cardiomyocytes were incubated with both a nuclear (Hoechst 33342 1 μM) and superoxide MitoSOX Red Mitochondrial superoxide indicator (ThermoFischer Scientific, M36008) (1 μM) for 10 to 15 min at the beginning of reoxygenation and imaged at 30 min of reoxygenation using a Zeiss Axio Observer Z1 inverted epifluorescence microscope with 63× oil objective lens and maintained at 37 °C throughout the experiment. The image system Zeiss Zen software was used for analyses.

### Statistical analysis

Values are expressed as mean ± standard error of the mean. Statistical significance was determined using one-way ANOVA. Significance was determined with the Bonferroni *post hoc* test. *p*-values <0.05 were considered significant. All statistical analyses employed GraphPad Prism Software (Version 5).

## Data availability

All data described are contained within the article. In addition, original data referred to in this article is held by the corresponding author's institution and will be shared upon reasonable request; e-mail: jseubert@ualberta.ca.

## Conflict of interest

The authors declare the following financial interests/personal relationships which may be considered as potential competing interests. A. K. is an employee, R. F. and W.-H. S. are co-founders of OMEICOS Therapeutics GmbH. J. M. S. received a collaborative research grant from OMEICOS Therapeutics GmbH. All other authors declared no competing interests for this work.
